# Theoretical basis for stabilizing messenger RNA through secondary structure design

**DOI:** 10.1093/nar/gkab764

**Published:** 2021-09-14

**Authors:** Hannah K Wayment-Steele, Do Soon Kim, Christian A Choe, John J Nicol, Roger Wellington-Oguri, Andrew M Watkins, R Andres Parra Sperberg, Po-Ssu Huang, Eterna Participants, Rhiju Das

**Affiliations:** Department of Chemistry, Stanford University, Stanford, CA 94305, USA; Eterna Massive Open Laboratory; Eterna Massive Open Laboratory; Department of Chemical and Biological Engineering, Northwestern University, Evanston, IL 60208, USA; Department of Biochemistry, Stanford University, Stanford, CA 94305, USA; Eterna Massive Open Laboratory; Department of Bioengineering, Stanford University, Stanford, CA 94305, USA; Eterna Massive Open Laboratory; Eterna Massive Open Laboratory; Eterna Massive Open Laboratory; Department of Biochemistry, Stanford University, Stanford, CA 94305, USA; Department of Bioengineering, Stanford University, Stanford, CA 94305, USA; Department of Bioengineering, Stanford University, Stanford, CA 94305, USA; Eterna Massive Open Laboratory; Eterna Massive Open Laboratory; Department of Biochemistry, Stanford University, Stanford, CA 94305, USA; Department of Physics, Stanford University, Stanford, CA 94305, USA

## Abstract

RNA hydrolysis presents problems in manufacturing, long-term storage, world-wide delivery and *in vivo* stability of messenger RNA (mRNA)-based vaccines and therapeutics. A largely unexplored strategy to reduce mRNA hydrolysis is to redesign RNAs to form double-stranded regions, which are protected from in-line cleavage and enzymatic degradation, while coding for the same proteins. The amount of stabilization that this strategy can deliver and the most effective algorithmic approach to achieve stabilization remain poorly understood. Here, we present simple calculations for estimating RNA stability against hydrolysis, and a model that links the average unpaired probability of an mRNA, or AUP, to its overall hydrolysis rate. To characterize the stabilization achievable through structure design, we compare AUP optimization by conventional mRNA design methods to results from more computationally sophisticated algorithms and crowdsourcing through the OpenVaccine challenge on the Eterna platform. We find that rational design on Eterna and the more sophisticated algorithms lead to constructs with low AUP, which we term ‘superfolder’ mRNAs. These designs exhibit a wide diversity of sequence and structure features that may be desirable for translation, biophysical size, and immunogenicity. Furthermore, their folding is robust to temperature, computer modeling method, choice of flanking untranslated regions, and changes in target protein sequence, as illustrated by rapid redesign of superfolder mRNAs for B.1.351, P.1 and B.1.1.7 variants of the prefusion-stabilized SARS-CoV-2 spike protein. Increases in *in vitro* mRNA half-life by at least two-fold appear immediately achievable.

## INTRODUCTION

Messenger RNA (mRNA) molecules have shown promise as vaccine candidates in the current COVID-19 pandemic (1–3) and may enable a large number of new therapeutic applications (4–6). However, a major limitation of mRNA technologies is the inherent chemical instability of RNA. mRNA manufacturing yields are reduced by degradation during *in vitro* transcription; mRNA vaccines stored in solution require *in vitro* stability, ideally over months under refrigeration (7); RNA vaccines deployed in developing regions would benefit from increased *in vitro* stability against high temperatures (8); and after being administered, mRNA vaccines require stabilization against hydrolysis and enzymatic degradation to sustain translation and immunogenicity in the human body (9).

**Figure 1.**
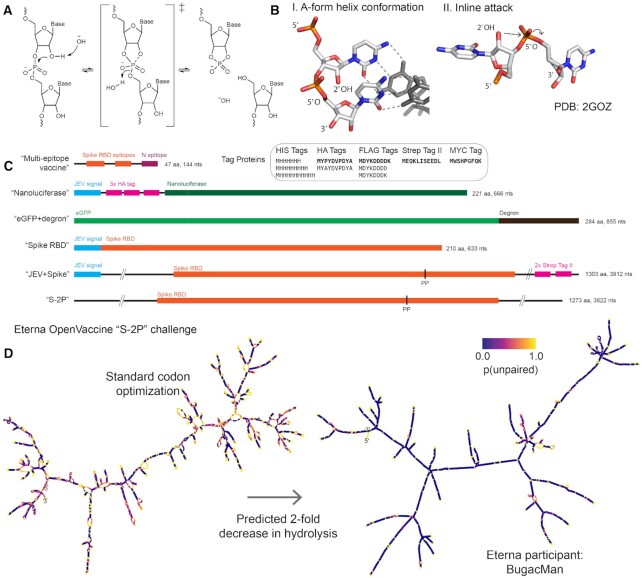
(**A**) Hydrolysis of the phosphodiester bond in the RNA backbone bond. This mechanism proceeds via an ‘inline attack’ backbone conformation, depicted in (**B**): the attacking 2′-hydroxyl group is in line with the phosphate group and the leaving 5′ oxygen. (**C**) Sequence schematics of all mRNA design challenges in this work. (**D**) mRNAs designed by conventional means for therapeutics are prone to hydrolysis in regions that have high probability of being unpaired (shown in yellow, left panel). A design for an mRNA vaccine encoding the prefusion-stabilized SARS-CoV-2 full spike protein (S-2P) dramatically reduces the probability of being unpaired throughout the molecule (purple, right panel).

RNA degradation depends on how prone the molecule is to in-line hydrolytic cleavage and attack by nucleases, oxidizers and chemical modifiers in the RNA’s environment (10–13). Amongst these degradation processes, in-line hydrolytic cleavage is a universal mechanism intrinsic to RNA. Cleavage of an RNA backbone phosphodiester bond is initiated by deprotonation of the 2′-hydroxyl group of the ribose moiety (14) (Figure 1A). The deprotonated hydroxyl group attacks the phosphate to form a pentacoordinate transition state. The formation of this transition state relies on the RNA backbone being able to adopt a conformation where the 2′- hydroxyl group is in line with the leaving 5′ oxyanion. The 5′ oxyanion then departs, leaving behind a 2′,3′-cyclic phosphate and a strand break in the RNA. The same mechanism underlies the action of self-cleaving ribozymes and protein-based nucleases, allowing this conformation to be characterized experimentally and visualized in crystal structures (Figure 1B, structure from (15)).

Hydrolysis sets a fundamental limit on the stability of mRNA medicines and technologies. The World Health Organization's target product profile for vaccines calls for formulations that remain effective after a month under refrigeration (2–8°C) (8). Deployment of mRNA vaccines for infectious disease outbreaks like the current COVID-19 pandemic would benefit from taking advantage of existing supply networks for conventional attenuated vector vaccines, which are set up for pre-filled syringes in saline buffer at near-neutral pH under refrigeration (8). However, model calculations of RNA hydrolysis as a function of pH, temperature, and ionic concentration (16), highlight potential problems for using the same supply networks for mRNA vaccines. Under refrigerated transport conditions (‘cold-chain’, 5°C, phosphate-buffered saline, pH 7.4, no Mg^2+^) (8), a naked RNA molecule encoding a SARS-CoV-2 spike glycoprotein, with a length of ∼4000 nucleotides in bulk solution would have a half-life of 900 days, with 98% intact after 30 days, fitting the target product profile for vaccines from the World Health Organization. However, a temperature excursion to 37°C is predicted to lead to a half-life reduced to 5 days, well under a month. Even if temperature can be maintained at 5°C, RNAs encapsulated in lipid formulations may be subject to increased hydrolysis if the lipid's cationic headgroups lower the p*K*_a_ of the ribose 2-hydroxyl group (17,18). If p*K*_a_ shifts as small as 2 units occur, the predicted half-life reduces from 900 days to 10 days, again well under a month (Table 1). Beyond the above considerations for a ∼4000 nt mRNA, the longer lengths of RNA molecules (>12 000 nt RNA) being considered for low-material-cost ‘self-amplifying’ mRNA (SAM) vaccines (3,19) are expected to exacerbate inline hydrolysis. In all conditions described above, the half-life for a SAM mRNA will be reduced by a further 3-fold compared to a non-SAM mRNA. As an example, if during storage or shipment at pH 7.4, the SAM vaccine of length 12 000 nts is subject to an excursion of temperature to 37°C for 2 days, the fraction of functional, full-length mRNA remaining after that excursion will drop to less than half of the starting RNA (Table 1). Beyond these calculations under storage and shipping conditions, an mRNA vaccine is expected to be highly unstable during *in vitro* transcription and upon delivery into the human body (half-lives reduced to hours due to presence of Mg^2+^ and physiological temperatures; Table 1). For these reasons, it is desirable to explore principles by which mRNA molecules might be designed to improve their stability against hydrolysis.

**Table 1.** Estimates for RNA degradation using the quantitative model presented by Li and Breaker (14) and the model for AUP presented in this work

Simulated condition (0.14 M [K^+^])	*T* (°C)	pH	[Mg^2+^] (mM)	RNA length (nucleotides)	AUP^a^	Cleavage rate per molecule (*k*_deg_) (10^–7^ min^–1^)	Half-life^b^ (days)
Refrigerated supply chain (‘cold chain’)	5	7.4	0	4000	0.4	5.1	941
Refrigerated supply chain, increased length (SAM RNA)	5	7.4	0	12 000	0.4	15.3	314
Refrigerated supply chain, pKa shifted by cationic formulation	5	9.4^c^	0	4000	0.4	470	10.2
Temperature excursion	37	7.4	0	4000	0.4	890	5.4
Manufacturing (*in vitro* transcription)^d^	37	7.6	14	4000	0.4	57 000	0.084
Physiological	37	7.4	1^e^	4000	0.4	2000	2.4

^a^Typical average unpaired probability (AUP) of 0.4 estimated from conventional design methods studied in this work.

^b^Calculated as *t*_1/2_ = $\ln 2/{k_{{\rm deg}}}.$

^c^Apparent pH at 2′ hydroxyl, simulating p*K*_a_ shift of 2 units induced by complexation with cationic lipid.

^d^(62), with pH 7.9 of Tris-HCl buffer corrected from 25°C to 37°C.

^e^(63).

One largely unexplored design method to reduce RNA hydrolysis that is largely independent of mRNA manufacturing, formulation, storage, and *in vivo* conditions is to increase the degree of secondary structure present in the RNA molecule. Hydrolysis has been found to be mitigated by the presence of secondary structure, which restricts the possible conformations the backbone can take and reduces the propensity to form conformations prone to in-line attack (20). Indeed, the technique of inline probing takes advantage of the suppression of in-line hydrolysis within double-stranded or otherwise structured regions to map RNA structure (21).

Here, we report a theoretical framework and computational results indicating that structure-aware design should enable immediate and significant COVID-19 mRNA vaccine stabilization. We present a principled model that links an RNA molecule's overall hydrolysis rate to base-pairing probabilities, which are readily calculated in widely used modeling packages (22–25). We define two metrics: the summed unpaired probability of a molecule, or SUP, and the average unpaired probability, or AUP, which is the SUP normalized by sequence length. By conducting computational design tests using a variety of protein targets (Figure 1C) and a variety of mRNA design methods, we provide evidence that both crowdsourced rational design on the Eterna platform and several optimization algorithms are able to appreciably minimize AUP for the coding sequence (CDS) across a range of mRNA applications. We compare these designs to solutions designed by minimizing the predicted folding free energy of the minimum free energy (MFE) structure, which can be achieved through an exact algorithm (26,27), and demonstrate that the two minimization problems are distinct and result in different constructs, both for small enumerable systems and larger systems of interest.

Our calculations predict that structure-optimizing designs can achieve at least two-fold increases in estimated mRNA half-life compared to conventional design methods (Figure 1D), independent of the mRNA length. Our results furthermore suggest that optimizing for mRNA half-life can be carried out while retaining other desirable sequence or structure properties of the mRNA, such as codon optimality, maximum stem length, and compactness measures, which may modulate *in vivo* mRNA translation and immune response. The predicted structures of these molecules are robust to a wide variety of perturbations, including temperature excursions, addition of flanking untranslated regions, and nonsynonymous changes in coding sequence, leading us to term them ‘superfolder’ mRNAs.

## MATERIALS AND METHODS

### Quantitative model for RNA hydrolysis

To predict the RNA degradation rates in Table 1, we used a model presented in ref. (14) for an inherent rate for phosphodiester bond cleavage as a function of pH, temperature, and ionic concentrations. The model is reproduced below:

$$\begin{eqnarray*} k_{{\rm projected}} &=& k_{{\rm background}} \times 10^{0.983({\rm pH} - 6)} \times 10^{- 0.24(3.16 -[{\rm K}^+]/1\, {\rm M})}\nonumber\\ &&\times 10^{0.07(T - 23^\circ {\rm C})} \times 69.3\frac{[{\rm Mg}^{2+}]^{0.80}}{1\,{\rm M}}\times 3.57\frac{[{\rm K}^{+}]^{-0.419}}{1\,{\rm M}}, \end{eqnarray*}$$

where ${k_{{\rm background}}} = \ 1.30\ \times {10^{ - 9}}$ min^–1^, which represents the model's selected reference point: pH 6, 23°C, [K^+^] = 3.17 M. The above equation was parametrized from measurements with [Mg^2+^] concentrations between 0.005 and 0.01 M; for conditions with 0 M Mg^2+^, we omit the last two terms.

### Eterna puzzle deployment

Eterna puzzles were launched in a series of rounds that gradually increased the complexity of the sequences designed and the information provided, as outlined in Supplementary Table S1. For all puzzles, the MFE structure calculated in the default folding engine is displayed as participants design the mRNA molecule. For full-length spike protein sequences, the algorithm LinearPartition-V (28) was used to calculate AUP. LinearPartition accelerates the base pair probability calculation by using a beam search approximation in the dynamic programming algorithm. The puzzle was set up for participants to design the full sequence at once.

### Generation of vendor-optimized sequences

The protein sequences for each target (Supplementary Table S2) were used to generate DNA sequences at Integrated DNA Technologies (IDT, https://www.idtdna.com/CodonOpt), Twist Biosciences (https://ecommerce.twistdna.com/app), and GENEWIZ (https://clims4.genewiz.com/Toolbox/CodonOptimization). For IDT and Twist Biosciences, multiple possible sequences were generated for a given protein sequence, while the GENEWIZ design tool returned one possible optimized DNA/RNA sequence per protein sequence.

### Stochastic minimization of AUP in RiboTree

A Monte-Carlo tree search algorithm named RiboTree was developed to stochastically minimize AUP for mRNA sequences. RiboTree uses the upper confidence bounds applied to trees (UCT) algorithm (29). The UCT loss function, as applied to the problem of sampling RNA sequences, is

(1) $$\begin{equation*}\frac{{{w_i}}}{{{n_i}}} + c\sqrt {\frac{{{\rm ln}\ {N_i}\ }}{{{n_i}}}} ,\end{equation*}$$

where ${w_i}$ is the total score considered for the node after the *i*th move, ${n_i}$ is the total number of times the node was visited after the *i*th move, and *c* is the exploration parameter, which determines the tradeoff between depth and breadth search. For minimizing AUP, moves consist of swapping synonymous codons and are accepted with a probability

(2) $$\begin{equation*}p\ \left( {i \to j} \right) = \ {e^{ - \beta \left( {{{\rm AUP}_j} - {{\rm AUP}_i}} \right)}},\end{equation*}$$

where AUP_i_ and AUP_j_ are the AUP values of states $i$ and $j$, respectively, and $\beta$ is a temperature parameter to control the acceptance rate. Runs were terminated after 6000 iterations. The RiboTree code is available for noncommercial use at https://eternagame.org/about/software.

### CDSFold

Solutions from CDSfold were obtained by running the CDSfold algorithm source code with varying maximum base-pairing distance. The CDSfold code is available at https://github.com/gterai/CDSfold or at https://github.com/eternagame/CDSfold_SU.

### LinearDesign

Solutions from LinearDesign were obtained using the LinearDesign server (http://rna.baidu.com/) and inputting the protein sequences given in Supplementary Table S2. A maximum beam size of 50 was used for prediction and the standard (Human) codon table.

### Metric calculations

Structure prediction and ensemble-based calculations were performed using LinearFold and LinearPartition with ViennaRNA and CONTRAfold parameters. Secondary structure features were calculated from predicted MFE structures using RiboGraphViz (www.github.com/DasLab/RiboGraphViz). The codon adaptation index (CAI) was calculated as the geometric mean of the relative usage frequency of codons along the length of the coding region, as described in (30):

(3) $$\begin{equation*}{\rm CAI}\ = \ {({\Pi _{i\ = \ 2}}^L{w_i})^{L - 1}};\ \ {w_i} = {f_i}\ /{\rm max}\left( {{f_j}} \right),\end{equation*}$$

where *f_j_* represents the frequency of all codons coding for amino acid at position *i*.

### Structure visualization

RNA secondary structures were visualized using draw_RNA (www.github.com/DasLab/draw_rna) and RiboGraphViz (www.github.com/DasLab/RiboGraphViz).

## RESULTS

### A biophysical model for RNA degradation

Previous studies have explored the design of mRNA molecules with increased predicted base-pairing (26,27), as evaluated by the predicted folding free energy of the mRNA’s most stable structure, but it is unclear if this metric is the correct one when improving stability of an RNA against degradation. Our computational studies are based on a principled model of RNA degradation that suggests an alternative metric. The rate at which an RNA is hydrolyzed is a property of the equilibrium probability that each nucleotide is in an unpaired state, leaving its 3′ phosphodiester linkage vulnerable to adopting the inline attack conformation (Figure 1B). A full derivation of this model, presented in Supporting Information, assumes that the degradation rate of a paired or unpaired nucleotide follows first-order kinetics (analogous to models developed for incorporating structure probing data (31–33)). We introduce the SUP as a readily calculated observable that is directly related to an RNA molecule's overall rate of cleavage. For an RNA molecule of length *N*, the SUP is defined as

(4) $$\begin{eqnarray*}{\rm{SUP}} = \sum\nolimits_i^N {{p_{{\rm unpaired}}}} (i) = \sum\nolimits_{i = 1}^N {\left[ {1 - \sum\nolimits_{j = 1}^N {p(i:j)} } \right]}.\nonumber\\ \end{eqnarray*}$$

The term ${p_{{\rm unpaired}}}( i )$ can be predicted in most widely-used RNA secondary structure prediction packages, which output base pair probabilities $p( {i:j} )$, the probability that bases *i* and *j* are paired.

The total rate of cleavage may be approximated as this measure, the sum of unpaired probabilities across all nucleotides of the RNA, multiplied by a constant ${k_{{\rm cleavage}}}^{{\rm unpaired}}$ that reflects the average cleavage rate of an unpaired nucleotide.

(5) $$\begin{equation*}{k_{{\rm cleavage}}}^{{\rm overall}} = {k_{{\rm cleavage}}}^{{\rm unpaired}}\ \times {\rm{SUP}}.\end{equation*}$$

We may also write Equation (5) in terms of the AUP, defined as

(6) $$\begin{equation*}{\rm{AUP}} = \frac{1}{N}\ \mathop \sum \limits_i^N {p_{{\rm unpaired}}}(i)=\frac{1}{N}\ {\rm{SUP}},\end{equation*}$$

which results in

(7) $$\begin{equation*}{k_{{\rm cleavage}}}^{{\rm overall}} = {k_{{\rm cleavage}}}^{{\rm unpaired}}\ \times N \times \ {\rm{AUP}}.\end{equation*}$$

Equation (7) makes explicit that the total rate scales with the sum of the unpaired probabilities of the RNA’s nucleotides – longer RNA molecules are expected to degrade faster in proportion to their length.

The AUP value is a number between 0 and 1 that reflects the overall ‘unstructuredness’ of the RNA, and accounts for unpaired regions in any secondary structure motif. Lower values correspond to lower probability of being unpaired, and therefore RNA molecules less susceptible to degradation. Under this model, it becomes possible to computationally study the question of how much an RNA might be stabilized if it is redesigned to form stable secondary structures, which we describe next.

### Small mRNA models reveal discrepancy in sequences optimized for SUP vs. sequences optimized for codon optimality or minimum folding free energy

To investigate the possible dynamic range in degradation lifetimes for mRNA, we started with mRNA design problems that were small enough to be tractable, i.e., all mRNA sequences that code for the target amino acid sequence could be directly enumerated and studied. We selected a collection of short peptide tags that are commonly appended or prepended to proteins to enable purification or imaging: His tags of varying lengths, human influenza hemagglutinin (HA) tag, Strep-tag II, FLAG fusion tag, and Myc tag sequences (34). We enumerated all the mRNA sequences that encode each protein. To estimate the possible range of improvement of stability that might be achieved through structure-award design, we calculated the ratio of AUP obtained between the average of conventionally designed mRNA sequences obtained from commercial vendor websites and the minimum AUP solution for tag proteins. We found fold-changes in AUP ranging from 1.27 to 2.23-fold, which, in our model, would correspond to significant increases in stability (Supplementary Table S3).

**Figure 2.**
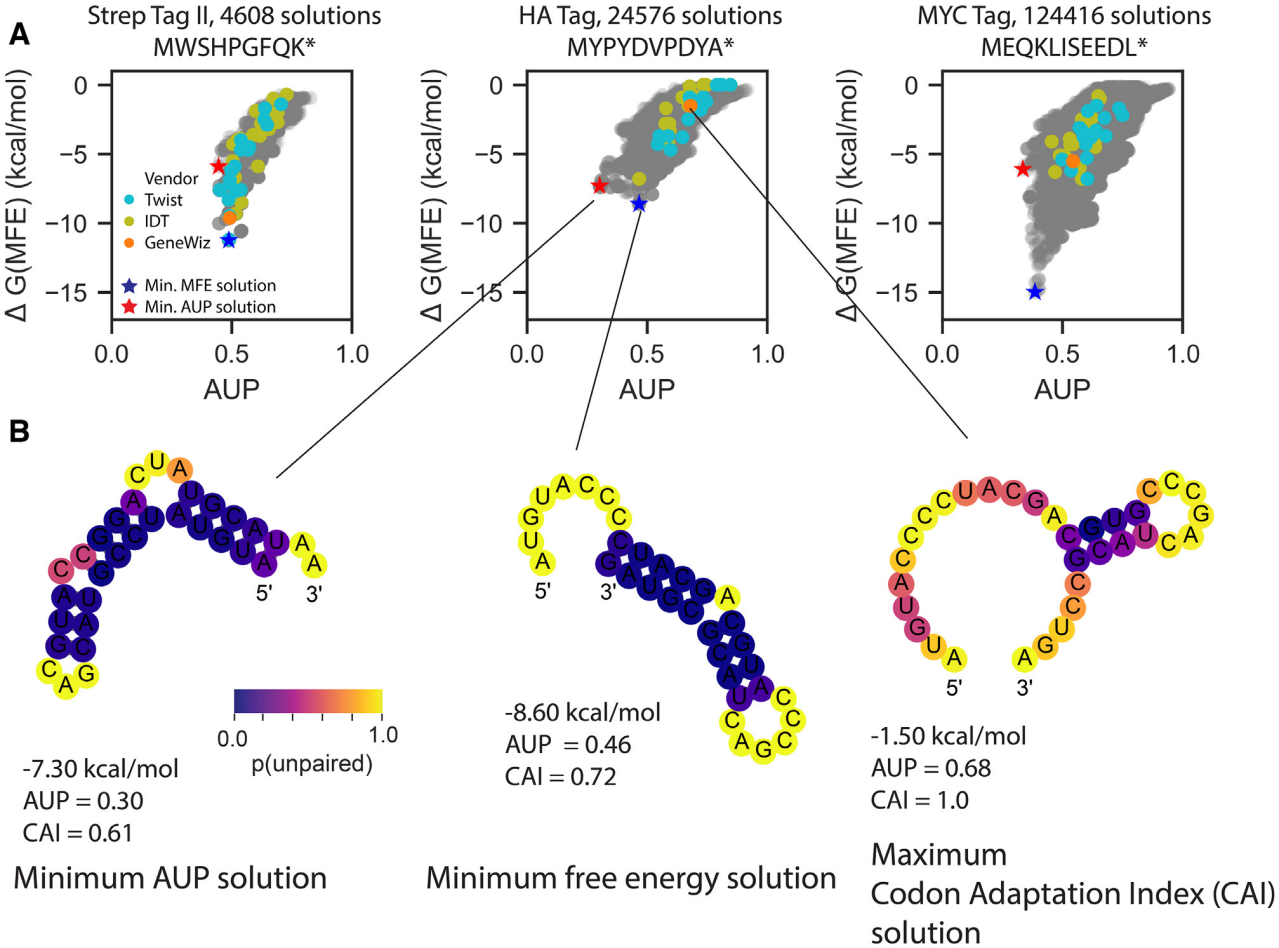
(**A**) Enumerating all possible coding sequences for small tag proteins reveals that the coding sequence with the lowest energy for its MFE structure (blue star) is not always the same as the coding sequence with the lowest AUP (red star). (**B**) MFE structures for mRNA solutions for the HA tag that minimize AUP, minimize free energy, and maximize codon adaptation index (CAI), with nucleotides colored by their computed probability of being unpaired.

We explored whether such stability improvements might be achieved with algorithms like LinearDesign and CDSfold, which optimize a different metric, the predicted folding free energy of the minimum free energy structure, Δ*G*(MFE) (26,27). For several model systems, the coding sequence with the lowest energy MFE structure was not the same as the solution with the lowest AUP (Figure 2A). Inspection of the two solutions clarifies why a structure with a higher free energy but a lower AUP would be preferred if we wish to reduce overall hydrolysis (Figure 2B): the minimal AUP solution for the HA tag has fewer ‘hot spots’ than the minimal free energy solution (7 versus 15 yellow nucleotides). Notably, the mRNA sequence maximizing the codon adaptation index (CAI) (30), often used to guide conventional mRNA design by codon optimization, is more unpaired. Evaluating AUP and Δ*G*(MFE) for these enumerated constructs with other secondary structure algorithms gave similar discrepancies between the minimum AUP and minimum Δ*G*(MFE) solutions (Supplementary Figure S1).

### A two-fold decrease in AUP is achievable for long mRNA constructs

To test the applicability of our insights from small peptide-encoding mRNA’s to more realistic protein-encoding mRNA design problems, we tested mRNA’s with lengths of hundreds of nucleotides encoding a variety of target proteins, some with therapeutic potential against SARS-CoV-2 and some commonly used in laboratory settings and animal studies to test protein synthesis levels (Figure 1C). The four systems were a multi-epitope vaccine design (MEV) derived from SARS-CoV-2 spike glycoprotein (S) and nucleocapsid (N) proteins; nanoluciferase; enhanced green fluorescence protein with an attached degron sequence (eGFP + deg), used by Mauger *et al.* (35) for characterizing mRNA stability and translation; and the SARS-CoV-2 spike receptor binding domain (RBD) of the SARS-CoV-2 spike protein. The protein targets of the mRNA design challenges are further described in Appendix B, and sequences are listed in Supplementary Table S2. Because enumeration of mRNA sequences is not possible for these problems, we compared sequences generated by a variety of methods: uniform sampling of codons (‘Uniform random codons’); uniform sampling of GC-rich codons only (‘GC-rich codons’); vendor-supplied servers from IDT, GENEWIZ and Twist; the algorithm CDSfold (26), which returns a sequence with minimal Δ*G*(MFE) solution; the algorithm LinearDesign, which returns a minimal Δ*G*(MFE) solution that is weighted by codon optimality, as well as sequences from other groups when possible (35). The algorithm CDSfold has the option to alter the maximum allowed base pair distance in the MFE structure of the final generated solution, allowing for solutions with a variety of global morphologies. We varied this parameter for each design challenge to test if AUP from CDSfold designs varied with maximal allowed base pair distance. We further developed a stochastic Monte Carlo tree search algorithm, RiboTree, to stochastically minimize AUP of model mRNAs (see Materials and Methods). We note that numerous algorithms have been developed to solve the problem of designing a RNA to fold to a particular structure (36,37), and some such as RNAiFold (38) allow for specifying codon constraints; however, these algorithms require specifying a desired target structure and cannot be directly applied here.

**Figure 3.**
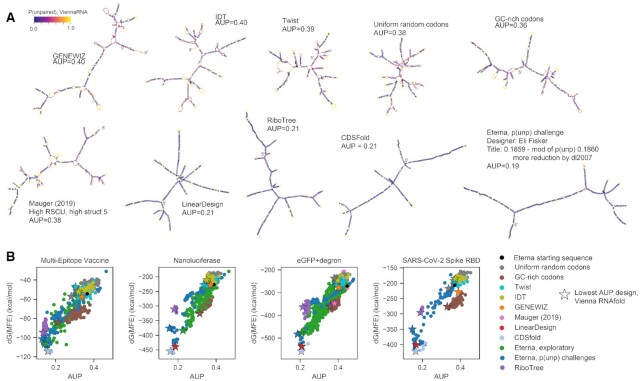
Sequences designed rationally by participants during Eterna's OpenVaccine challenge result in the lowest AUP values for mRNAs encoding a variety of model proteins used for studying translation and as model vaccines, ranging in length from 144 nucleotides (the Multi-epitope Vaccine) to 855 nucleotides (eGFP + degron (35)). (**A**) Force-directed graph visualization of MFE structures predicted for sequences with lowest AUP value from each design source, colored by the computed probability of each nucleotide being unpaired. (**B**) While Δ*G*(MFE) and AUP are correlated, the design with the lowest AUP is not the same as the design with the lowest Δ*G*(MFE). Starred points indicate the design for each design strategy with the lowest AUP value, calculated with ViennaRNA.

**Figure 4.**
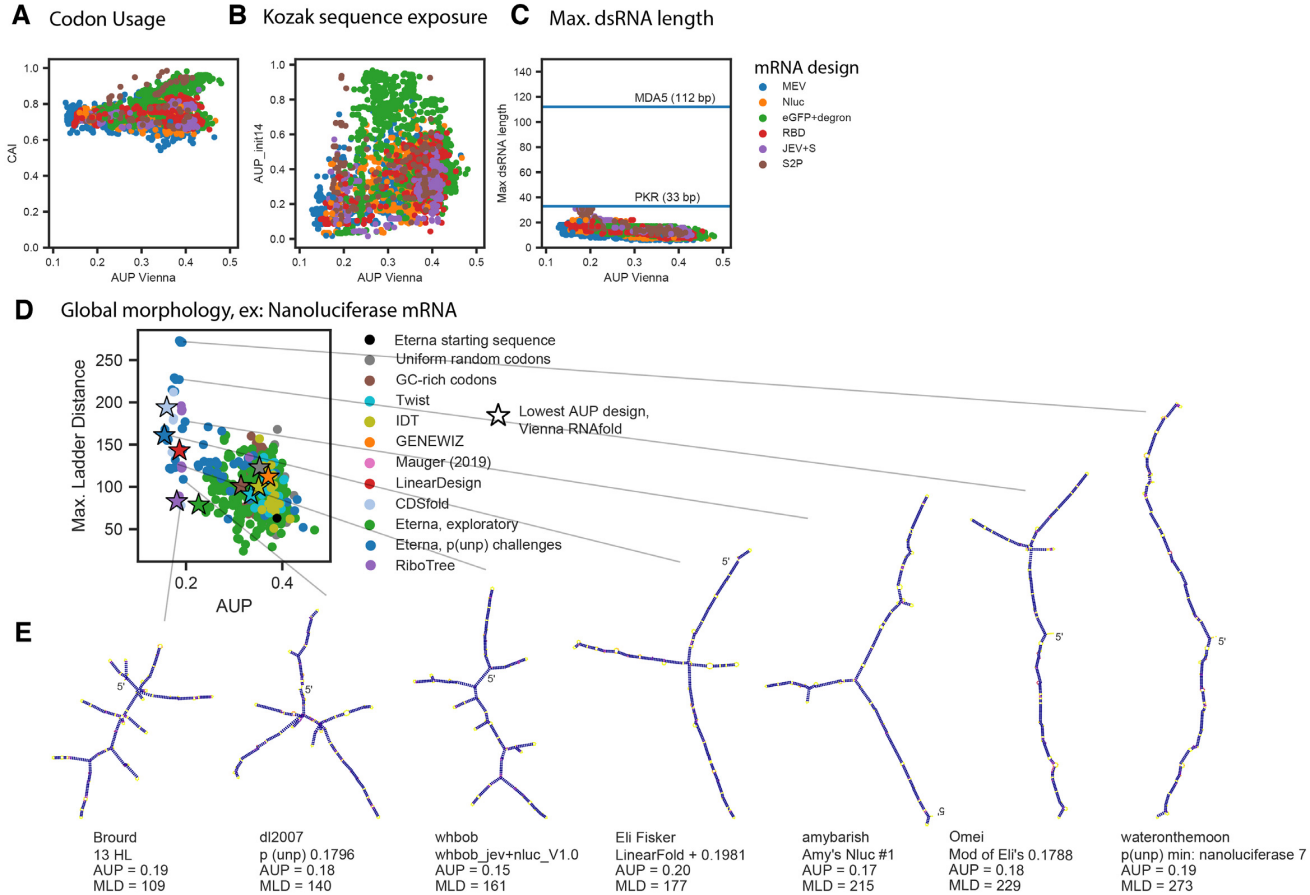
mRNA designs with low AUP (**A**) have codon adaptation index values consistent with high translation efficiency, (**B**) show a range of values for the probability the first 14 nucleotides of the coding sequence is unpaired (AUP^init,14^), and (**C**) do not have helices longer than 33 nts, suggesting they are unlikely to raise an innate immune response that would shut down cellular mRNA translation. (**D**) Eterna designs show structural diversity as characterized by the maximum ladder distance (MLD), the longest path of contiguous helices present in the minimum free energy (MFE) structure of the molecule. (**E**) MFE structures predicted in the ViennaRNA structure prediction package are depicted for designs with a variety of MLD values, indicating similarly stabilized stems for a range of topologies.

In addition, we crowdsourced solutions through the online RNA design platform Eterna (39). 112 participants (screen names listed in Supplementary Table S4) contributed a total of 3482 solutions over seven rounds spanning 23 March 2020 to 2 January 2021 (Supplementary Table S1). Participants discussed solutions and strategies in online forums, and sometimes worked cooperatively by making modifications to other participants’ solutions. Players also utilized the LinearDesign web server to obtain initial stabilized structures for portions of puzzles. Early Eterna challenges, labeled ‘Eterna, exploratory’ in Figures 3 and 4, were not set up with any specific optimization targets other than a general call to create mRNAs that coded for the target proteins but formed significant structures in Eterna's game interface, which provides folding calculations in a number of secondary structure prediction packages (see Materials and Methods). An additional set of Eterna sequences were solicited in the ‘p(unp) challenges’, where the AUP metric was calculated and provided to Eterna participants within the game interface to guide optimization.

For all four challenges, the sequences with the lowest AUP values were designed by Eterna participants. We found that designs from the tested algorithmic and crowdsourcing approaches encompassed a wide range of sequence space, as monitored by principal component analysis (PCA), pairwise Levenshtein distance, and positional entropy, and that sequences with low AUP values did not localize to specific regions of sequence space (Supplementary Figure S2). Figure 3A depicts MFE structures of the minimal AUP sequence for each design method for the eGFP + degron challenge (the longest mRNA), with nucleotides colored by their unpaired probability, as calculated in the ViennaRNA folding package (22). MFE structures of minimal AUP sequences from each mRNA challenge are in Supplementary Figure S3. Structures portrayed in Figure 3A indicate visual hallmarks of structures with lower AUP: solutions from LinearDesign, CDSFold, RiboTree and Eterna have longer helices, fewer loops and junctions, and lower unpaired probabilities in stems (indicated by dark purple). Notably, the solutions with the minimal AUP were distinct from solutions with the lowest Δ*G*(MFE) (Figure 3B) for all four challenges. Table 2 contains summary statistics for AUP values for design methods separated by standard methods (codon sampling, gene vendor tools) and methods intended to stabilize secondary structure (Eterna AUP rational design, CDSfold, LinearDesign, RiboTree).

**Table 2.** Statistics of AUP values obtained in comparing different classes of design methods on mRNA design challenges in this study.

mRNA design challenge	Multi-epitope vaccine	Nano-luciferase	eGFP + degron	spike RBD	JEV + spike	S-2P
Protein length (aa)	47	221	284	210	1303	1273
mRNA CDS length (nt)	144	666	855	633	3912	3822
Standard methods^b^, mean AUP (standard deviation in parentheses)	0.34(4)	0.36(2)	0.40(2)	0.39(2)	0.40(2)	0.40(2)
Stabilizing methods^c^, mean AUP (standard deviation in parentheses)	0.15(2)	0.17(2)	0.20(1)	0.17(2)	0.18(2)	0.19(2)
Stabilizing methods^c^, min. AUP^d^	0.13	0.15	0.19	0.15	0.17	0.17
Mean standard / min stabilized	2.6	2.4	2.1	2.6	2.4	2.4

^a^mRNA length = 3 ⋅ protein length + 3 (stop codon).

^b^Uniform codons, GC codons, GENEWIZ, IDT, Twist.

^c^Eterna p(unp) challenge, RiboTree, CDSfold, LinearDesign.

^d^In all cases, minimal AUP was achieved in Eterna p(unp) challenge.

The values of AUP achieved by Eterna participant submissions in the ‘p(unp) challenge’ (mean and standard deviations of MEV: 0.22 ± 0.08, Nluc: 0.24 ± 0.08, eGFP: 0.28 ± 0.08, spike RBD: 0.24 ± 0.08) were significantly lower than values from standard methods, including codon random sampling and vendor-generated sequences (MEV: 0.34 ± 0.04, Nluc: 0.36 ± 0.02, eGFP: 0.40 ± 0.02, spike RBD: 0.39 ± 0.02, Table 2). The lowest AUP values from Eterna participants (MEV: 0.128, Nluc: 0.155, eGFP: 0.186, spike RBD: 0.148) were lower in each case than the AUP values of LinearDesign constructs, (MEV: 0.159, Nluc: 0.186, eGFP: 0.208, spike RBD: 0.167), CDSfold constructs (MEV: 0.160, Nluc: 0.160, eGFP: 0.206, spike RBD: 0.165), or of minimum AUP solutions from RiboTree (MEV: 0.134, Nluc: 0.181, eGFP: 0.214, spike RBD: 0.190). RiboTree came closest (within 5%) to the minimal Eterna AUP value for the shortest mRNA sequence, suggesting that RiboTree was better able to search sequence space for the shorter sequences.

One of the challenges, the eGFP + degron mRNA, could be compared to designs developed by Mauger *et al.* based on folding free energy optimization (35). The minimal AUP value from those sequences (0.381) was similar to the value obtained from randomly sampled codons, indicating that explicit optimization of AUP rather than folding free energy is necessary for applications seeking stability against hydrolysis. Repeating these analyses of mRNA AUP based on other secondary structure packages (25) reveals similar results in fold-change and relative ranking of designs (Supplementary Figure S4).

We were interested to note that RiboTree solutions exhibited low AUP while not necessarily minimizing Δ*G*(MFE). Minimum AUP solutions from RiboTree had Δ*G*(MFE) values that were up to 25% greater (less stable) than Δ*G*(MFE) values of minimum Δ*G*(MFE) solutions, which came from Eterna participants (MEV: 7%, nanoluciferase: 12%, eGFP + deg: 25%, spike RBD: 16%). Although Δ*G*(MFE) is tractable as a metric to minimize in dynamic programming methods, it only represents the stability of one structure in the structure ensemble of a molecule. We therefore also compared AUP to the free energy of the full structure ensemble (Δ*G*(ensemble)) for all constructs, and found similarly that constructs minimizing AUP did not minimize Δ*G*(ensemble) (Supplementary Figure S5). Minimizing AUP without minimizing either Δ*G*(MFE) or Δ*G*(ensemble) may prove to be a valuable design strategy for developing mRNAs that are stable under storage but need to be sufficiently unstable as to exhibit cooperative unfolding by the cells’ translational apparatus.

#### Diversity of properties related to translation and immunogenic function

After establishing the feasibility of designing mRNA sequences with reduced AUP, we wished to determine if these sequences might be viable for translation and for either preventing or eliciting innate immune responses. In advance of experimental tests, we tabulated sequence and structure properties that have been hypothesized to correlate with translation and immunogenicity.

We first characterized the CAI (30) of sequences across design methods, as this measure has been implicated in improving translation efficiency (40–44). We found that across all mRNA design challenges, minimal AUP sequences consistently had CAI values greater than 0.7 (Figure 4A). Another design feature that has been hypothesized to influence protein translation efficiency is the exposure of the CDS immediately upstream of the initiation codon (45,46). We calculated the average unpaired probabilities of the first 14 nucleotides (45), termed AUP^init,14^, in the presence of our model UTRs from human hemoglobin subunit beta (HBB). A higher value of AUP^init,14^ indicates a more exposed ribosome initiation site, and is expected to correlate with higher translation efficiency. We found a range of AUP^init,14^ values possible for low AUP sequences (Figure 4B). These analyses suggest that it is feasible to design low AUP sequences that are translatable, as assessed by the available metrics of CAI and AUP^init,14^.

Another important consideration is the possibility of mRNA therapeutics eliciting immunogenic responses from pathways that recognize double-stranded RNA helices (47–49). We found that none of the sequences characterized included Watson-Crick helices longer than 33 base pairs, a measure that has been found to be the minimum length that leads to global shutdown of cellular mRNA translation after sensing by protein kinase R (PKR) (50), nor longer than 112 bp, a length observed to stimulate cooperative binding of the Retinoic acid-inducible gene I (RIG-I)-like receptor melanoma differentiation-associated protein 5 (MDA5) (48) (Figure 4C). However, PKR has been characterized to bind dsRNA containing bulges and mismatches one or two nucleotides long within ∼2-fold affinity of A-form dsRNA (51). We therefore also calculated the maximum dsRNA length for all designed constructs considering single- and di-nucleotide bulges and 1 × 1 and 2 × 2 nucleotide internal loops (Supplementary Figure S6). When considering these defects, many of the low-AUP constructs have maximum dsRNA lengths greater than 33 bp, yet still shorter than 112 bp. It may be possible for dsRNA-binding proteins to bind these constructs with reduced affinity if synthesized with unmodified nucleotides. In general, the diversity of maximum dsRNA lengths achievable for low-AUP constructs suggests that a less drastic innate immune response might be achieved and the response may be tunable depending on whether such responses are desirable (mRNA vaccines) or not (e.g. for anti-immune mRNA therapeutics).

Finally, the sequences designed in the above challenges did not contain UTRs. We compared the AUP of the above designs in the presence of HBB UTRs, as well as AUP^init,14^, as the presence of a 5′ UTR could base pair with a ribosome binding site. We found that for the collected sequence designs, the AUP calculated in the context of HBB UTRs had high correlation to the AUP of the CDS only (MEV: 0.91, nanoluciferase: 0.98, eGFP + degron: 0.99, spike RBD: 0.99, Supplementary Figure S7, UTR sequences in Supplementary Table S2). This indicates that the overall AUP of a designed CDS also maintains low AUP in the context of UTRs. The correlation between AUP^init,14^ in the absence and in the presence of UTRs was less robust (MEV: 0.32, nanoluciferase: 0.57, eGFP + degron: 0.56, spike RBD: 0.71, Supplementary Figure S7), but still suggests that constructs may be designed to maintain high AUP^init,14^ that is robust to adding UTRs.

In addition to structural characteristics that affect stability against *in vitro* hydrolysis, translatability and degradation rates in cells, and immunogenicity of mRNA molecules, we expect there are many structural characteristics that relate to a molecule's *in vivo* persistence that are not yet well understood. The ability to design multiple low-AUP sequences with a large range of alternative structures increases the potential that a functional design may be found in empirical tests or as the connections between mRNA structure and function are better understood. For instance, in Figure 3A, we observed that although LinearDesign, RiboTree, and Eterna sequences for an eGFP + degron mRNA all have AUP values within 10% of each other, they have different secondary structures. The same can be seen for all the mRNA design problems we tested (Supplementary Figure S3).

As a quantitative evaluation of structural diversity, we characterized the maximum ladder distance (MLD) of designed sequences. This measure has been used to describe the compactness of viral genomic RNAs and has been hypothesized to be relevant for viral packaging, immunogenicity, and biological persistence (52,53). If an RNA molecule's secondary structure is represented as an undirected graph, where edges represent helices, edge lengths correspond to helix lengths, and vertices correspond to loops, the MLD is the longest path that can be traced in the graph. Genomic viral RNAs have been demonstrated to have shorter MLDs than equivalent random controls, and molecules with shorter MLDs have been shown to be more compact experimentally, a feature that may also contribute to persistence (41). We found that AUP and MLD were negatively correlated across the MEV, nanoluciferase, eGFP + degron, and spike RBD challenges (Pearson correlation coefficients of −0.64, −0.59, −0.62, −0.70, respectively, nanoluciferase values in Figure 4D, all challenges in Supplementary Figure S8). This overall (negative) correlation reflects how minimizing AUP leads to larger average MLD values. Nevertheless, we note that the MLD values still fall over a wide range for sequences with low AUP. Example structures from the nanoluciferase challenge, depicted in Figure 4E, range from highly branched, compact structures (Figure 4E, left) to long, snake-like structures (Figure 4E, right). These structures exhibit uniformly low unpaired probabilities in stems (indicated by dark purple coloring), with the main difference being the layout of stems. In addition to MLD, we calculated several other metrics characterizing structure, such as counts of different types of loops and junctions, the ratio of number of hairpins to number of 3-way junctions in the MFE structure, introduced in ref. (52) as a measure of branching, and mean distance between nucleotides in base pairs. In all cases, values ranged by over 2-fold in low-AUP solutions, underscoring the diversity of structures that can be achieved. The diversity of these structural metrics, as well as purely sequence-based metrics that may affect mRNA function, like dinucleotide frequency (54–56), are illustrated in Supplementary Figure S9.

These results demonstrate that both automated and rational design methods are capable of finding RNA sequences with low AUP values but a wide range of diverse structures. Testing these mRNAs experimentally for their translation rates and persistence in cells and in animals may help address the relationship between MLD and mRNA therapeutic stability.

### Eterna participants are able to design stabilized SARS-CoV-2 full spike Protein mRNAs

For longer mRNA design problems, including the SARS-CoV-2 spike protein mRNA used in COVID-19 vaccine formulations (3822 nts), we noted that the computational cost associated with computing thermodynamic ensembles associated with AUP became slow and hindered automated or interactive design guided by AUP. We therefore sought other observables that were more rapid to compute to guide design of RNA’s stabilized against hydrolysis. We calculated correlations between many observables and AUP (Supplementary Figure S10), and found that for all four challenges, the number of unpaired nucleotides in the single MFE structure was the most correlated with AUP, giving near-perfect correlations (0.98, 0.99, 0.99, 0.99, respectively). We leveraged this observation to launch another design puzzle on Eterna: minimizing the number of unpaired nucleotides in the MFE structure, as a proxy for AUP, for a vaccine design that includes the full SARS-CoV-2 spike protein (‘JEV + spike’). We found that Eterna participants were capable of finding values for AUP as low as in previous challenges, despite the fact that the JEV + spike mRNA was over four times as long as previous challenges. Again, this solution was distinct from the minimal Δ*G*(MFE) design calculated in CDSFold (Supplementary Figure S5). The lowest AUP value for the JEV + spike protein was 0.17, 2.4-fold lower than the minimum AUP values from conventional design methods (0.40 ± 0.02). To test whether similar optimization could be achieved with automated methods, we ran RiboTree to minimize the number of unpaired nucleotides. The larger size of the JEV + spike protein meant that it took longer for RiboTree to minimize the solution. Starting from a random initialization and running RiboTree for 6000 iterations (2 days) resulted in a construct with an AUP of 0.254. Seeding RiboTree with a starting sequence partially stabilized using the LinearDesign server (AUP: 0.212) resulted in an AUP (0.206), the lowest achieved by an automated approach but still higher than than the best AUP achieved on Eterna (0.166).

**Figure 5.**
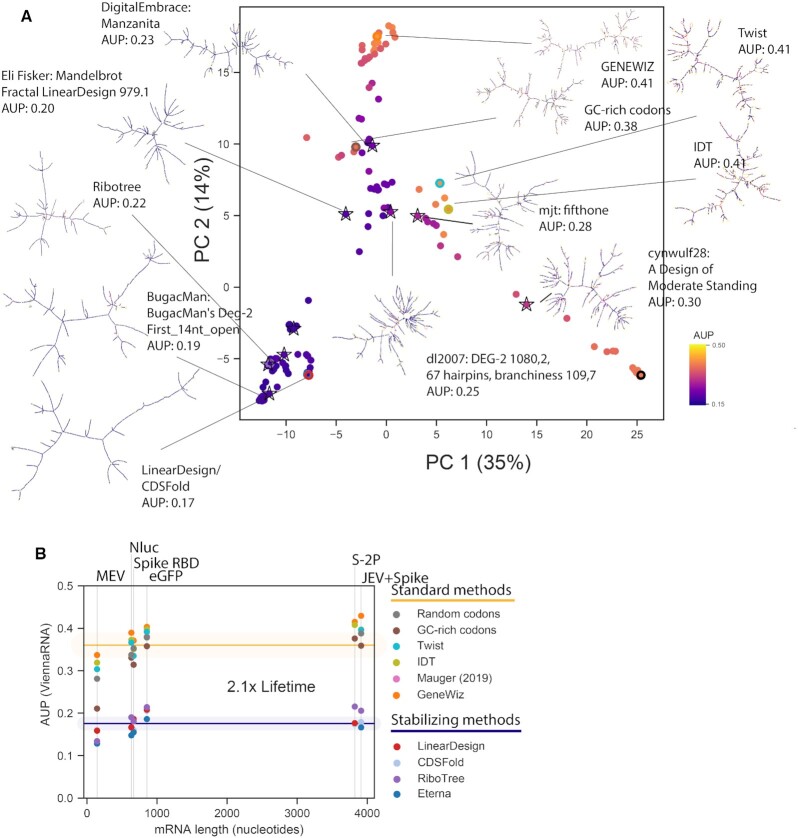
Design of stabilized mRNAs for the SARS-CoV-2 full spike protein achieve the same degree of stabilization as in smaller mRNA design challenges. (**A**) Solutions voted upon by the Eterna community show a diversity of structures while maintaining low AUP values. The solutions with the lowest AUP are structurally similar to and were derived from the Δ*G*(MFE) optimal structure from LinearDesign. (**B**) AUP values from different design methods are consistent across different mRNA lengths. A two-fold increase in lifetime is predicted by changing from a ‘Standard’ design method (methods that do not stabilize structure) to a design method that increases structure.

The SARS-CoV-2 spike protein sequence used in most vaccine formulations has been a double proline mutant S-2P that stabilizes the prefusion conformation of the 1273-aa spike (57). We launched a final puzzle on Eterna calling for solutions for stabilized mRNAs encoding the S-2P protein. Participants were provided with a variety of metrics of both predicted stability and structure (Supplementary Figure S11, Materials and Methods), and were not specifically asked to optimize any of the metrics. Out of 181 submissions, the top 9 solutions that were voted upon demonstrated a diverse set of sequences, some prioritizing structure diversity, some prioritizing high stability, all demonstrating low AUP values (Figure 5A). As with shorter mRNAs, S-2P solutions with the lowest AUP values—from Eterna participants, RiboTree, CDSfold, and LinearDesign—demonstrate a 2-fold reduction in AUP from mRNAs designed through randomly selecting codons or from codon optimization algorithms from gene synthesis vendors (Figure 5B).

#### Highly stable mRNAs are robust to variations in design

**Figure 6.**
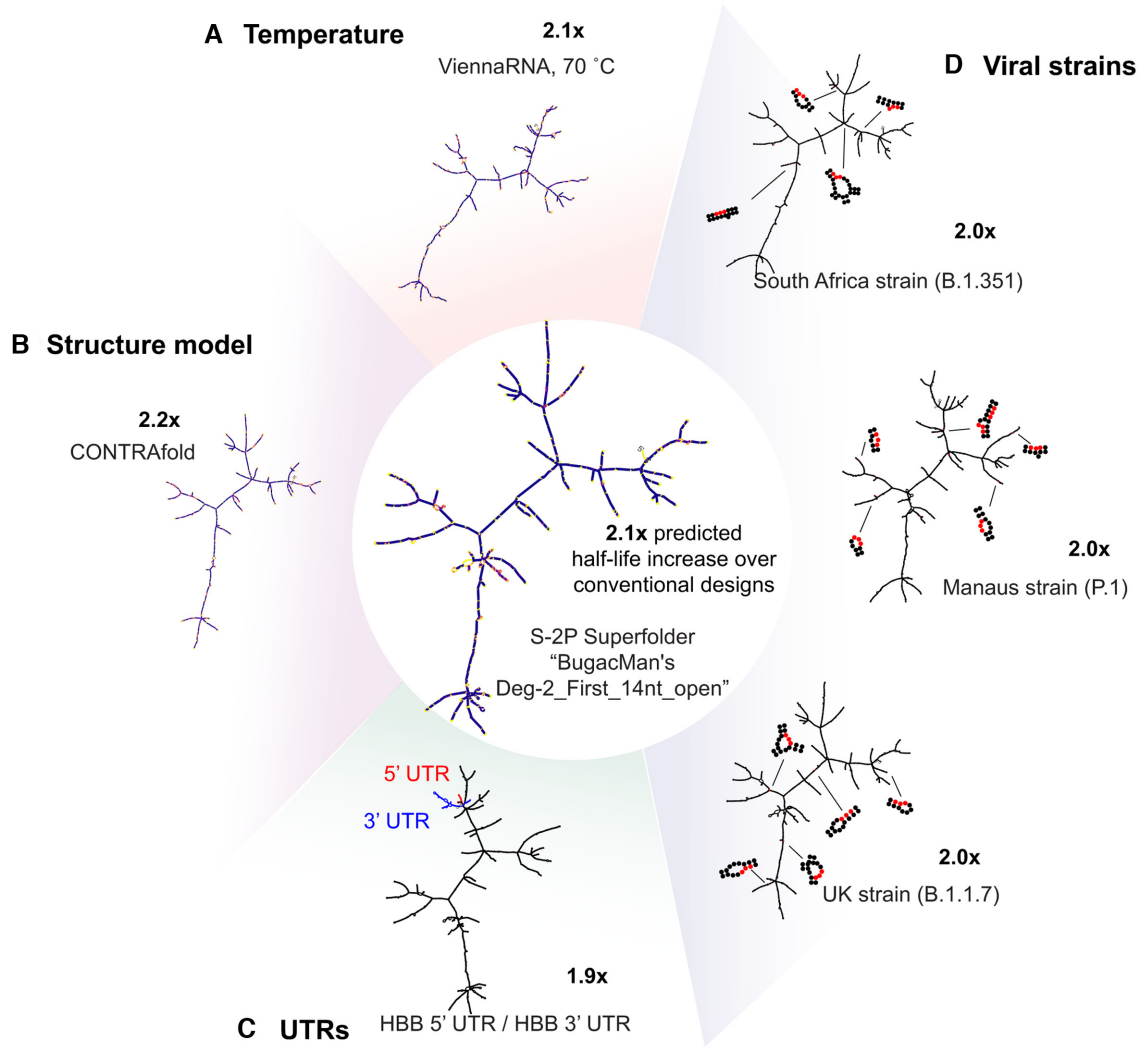
Low AUP solutions as superfolder mRNAs. Predicted stabilization derived from a low AUP solution is robust to (**A**) when calculated at higher temperatures, (**B**) when calculated in other folding algorithms, (**C**) in the presence of added UTRs and (**D**) small variations in protein sequence.

There are several design contexts in which it would be advantageous to adapt an existing highly stable mRNA design, rather than design an mRNA *de novo*. These include changes in environment (e.g. higher temperature), changes to protein sequence, potentially needed to rapidly develop booster vaccines for variant strains (58), as well as altering the UTRs used, which would allow for flexibility in testing different expression formulations post-mRNA-design. We tested the robustness of designed S-2P mRNA stability to small changes in protein sequence and to different UTRs for a subset of the S-2P sequences collected. We selected the 9 top-voted sequences from the Eterna S-2P round, as well as one representative sequence from other methods (Twist, IDT, GENEWIZ, GC-rich, LinearDesign and RiboTree). Predicted structures and predicted fold-change over conventional mRNA design methods are presented in Figure 6 for the example mRNA design ‘BugacMan's Deg-2_First_14nt_Open’ and in Supplementary Figure S12 for analogous predictions for a second design ‘Eli Fisker LinearDesign Mandelbrot Fractal’.

As a first test of design robustness to destabilizing environments, we compared the predicted AUP at the default temperature of our folding packages (37°C) to the predicted AUP at 70°C. Sequences with low AUPs maintained low AUP predicted at 70°C, while sequences with high AUPs had AUPs raised by roughly an additional 30% (Figure 6A, Supplementary Figure S13). The same global folds and amount of stabilization over conventionally-designed mRNAs were also predicted in CONTRAfold (25) (Figure 6B).

We additionally tested the robustness of the structured mRNA designs to sequence changes. To test the effect of adding UTRs, we calculated AUP for the subset of sequences in the context of HBB 5′/3′ UTR. The AUP of the full constructs exhibited very high correlation to the AUP of the CDS only (0.999). Importantly, highly structured sequences also retained very similar low AUP values upon adding UTRs (Figure 6C). To test robustness of folding stability with respect to nonsynonymous coding changes, we tested a simple heuristic to ‘hot fix’ an mRNA design to code for a new protein variant: for each amino acid mutation, we replaced the new codon with the most GC-rich codons for the mutant. We used this heuristic to design mRNA sequences coding for S-2P antigens appropriate strains B.1.1.7 (59), P.1. (59), and B.1.351 (59), which present 10, 10, and 12 amino acid changes compared to S-2P, respectively (protein sequences listed in Supplementary Table S2). We found that for the mRNAs tested, the AUP of the modified mRNA had near-perfect correlation with the AUP of the original mRNA (0.999, 0.996, 0.999, for B.1.1.7, P.1, B.1.351, respectively). The addition of mutations did not perturb the global layout of the mRNA design (Figure 6D). Taken together, these results indicate that for messenger RNAs of similar length to the SARS-CoV-2 spike protein (3822 nts), predicted increases in stability are robust to protein sequence mutations and changes in UTRs.

## DISCUSSION

In this work, we have developed a framework for stabilizing messenger RNA against *in vitro* hydrolysis through structure-based design. We presented a model relating the degradation rate of an RNA molecule to the AUP, a measure readily calculated in available secondary structure prediction packages. We calculated AUP across a large collection of messenger RNA designs for small peptides, for reporter proteins like eGFP and nanoluciferase, and for antigens under consideration for SARS-CoV-2 vaccines. The solutions with the lowest AUP values—from Eterna participants, the new RiboTree algorithm, and LinearDesign—demonstrate a 2-fold reduction in AUP from mRNAs designed through randomly selecting codons or from codon optimization algorithms from gene synthesis vendors (Figure 5B). A 2-fold reduction in AUP corresponds to a 2-fold increase in mRNA half-life, a potentially significant improvement in the context of mRNA biotechnology and current logistical challenges facing mRNA vaccine distribution for the COVID pandemic: extra days or weeks of stability of COVID mRNA vaccines in aqueous buffer could dramatically increase the number of people who can receive doses and potentially obviate the need to ship the vaccines in frozen vials (8). Parallel work in experimentally testing the predicted increase in half-life is ongoing (60).

Important for practical applications, mRNAs stabilized through designed secondary structure must remain functional in terms of producing protein in human cells and in terms of giving a controlled immune response. Experimental tests of protein production in human cells are ongoing (60). The immunogenicity of mRNA molecules remain difficult to predict from sequence and structure, so it is important that when designing for one property (i.e. low hydrolysis, as investigated in this work), a wide range of values for other sequence and structure features should be achievable. We were encouraged to find that low AUP designs do indeed encompass a variety of structures, as measured by maximum dsRNA length, maximum ladder distance, number of multiloop junctions and numerous other properties. Importantly, if mRNA design efforts require maximizing or minimizing these sequence or structural metrics, e.g., to enhance packaging into lipid nanoparticles or to suppress innate immune responses, both Eterna crowdsourcing and the automated RiboTree framework allow for optimization of such properties simultaneously with AUP. Finally, we provide computational evidence that for mRNA designs at the length scale of the SARS-CoV-2 spike protein, stabilized mRNA designs are robust to a number of changes: increasing the temperature, altering the protein sequence (potentially useful for developing ‘booster’ vaccines for variant strains), and adding different UTR sequences to the designed CDS. By analogy to ‘superfolder’ proteins that are stabilized against similar perturbations of environment and sequence (61), we propose to call these sequences ‘superfolder’ mRNAs.

Further increases in mRNA lifetime through structure-guided design are likely possible, as the computational model underlying our study is expected to underestimate how much stabilization is achievable in practice. In particular, some secondary structure and sequence motifs may be less prone to hydrolysis than others (11,16,20). Knowledge and prioritization of those specially hydrolysis-resistant motifs in mRNA designs could lower degradation rates beyond those achieved in the present study. Critical for achieving further improvements in stability in practice will be collection of large experimental data sets mapping hydrolysis rates of many RNAs at single nucleotide resolution, and predictive models of hydrolysis rates trained on such data sets. Measurements of protein expression from superfolder mRNAs will also be important to test compatibility with cellular translation and sustained or increased cellular lifetime, as has been recently observed (35,60). We propose that with such empirical knowledge, mRNA lifetimes in storage and shipping may be extended by much more than two-fold with maintained or enhanced function.

## DATA AVAILABILITY

The OpenVaccine sequences and calculated features are included in the supplementary information of this manuscript. The same data, as well as scripts to reproduce analysis, are available in the ‘OpenVaccine-solves’ database under an Open COVID license at https://eternagame.org/about/software.

## Supplementary Material

gkab764_Supplemental_Files

## Notes

**Dedication**: This paper is in memory of Malcolm Watson, one of Eterna's most active participants and longtime contributors.
