# Correction to ‘Theoretical basis for stabilizing messenger RNA through secondary structure design’

**DOI:** 10.1093/nar/gkab911

**Published:** 2021-09-30

**Authors:** Hannah K Wayment-Steele, Do Soon Kim, Christian A Choe, John J Nicol, Roger Wellington-Oguri, Andrew M Watkins, R Andres Parra Sperberg, Po-Ssu Huang, Eterna Participants, Rhiju Das

**Affiliations:** Department of Chemistry, Stanford University, Stanford, CA 94305, USA; Eterna Massive Open Laboratory; Eterna Massive Open Laboratory; Department of Chemical and Biological Engineering, Northwestern University, Evanston, IL 60208, USA; Department of Biochemistry, Stanford University, Stanford, CA 94305, USA; Eterna Massive Open Laboratory; Department of Bioengineering, Stanford University, Stanford, CA 94305, USA; Eterna Massive Open Laboratory; Eterna Massive Open Laboratory; Eterna Massive Open Laboratory; Department of Biochemistry, Stanford University, Stanford, CA 94305, USA; Department of Bioengineering, Stanford University, Stanford, CA 94305, USA; Department of Bioengineering, Stanford University, Stanford, CA 94305, USA; Eterna Massive Open Laboratory; Eterna Massive Open Laboratory; Department of Biochemistry, Stanford University, Stanford, CA 94305, USA; Department of Physics, Stanford University, Stanford, CA 94305, USA

The authors wish to correct the supplementary data in their article (1).

The authors had included three supplementary files for peer-review purposes that should not have been published with the article. The three files have been removed from the supplementary data.
