# Supplementary material for: Theoretical basis for stabilizing messenger RNA through secondary structure design
Source: Nucleic Acids Res. 2021 Sep 14;49(18):10604–17. doi: 10.1093/nar/gkab764 (PMC8499941; doi:10.1093/nar/gkab764)
Supplement: gkab764_Supplemental_Files [file gkab764_supplemental_files.zip › Supplementary table legends.docx]

**Supplementary table legends**

**Table S1. Details of Eterna Lab Rounds**

**Table S2: Protein sequences used for mRNA design challenges.**

**Table S3. AUP values for example tag proteins and calculated fold-change decrease in AUP from standard design methods to the global minimum AUP value.**

**Table S4: Eterna usernames of contributors to OpenVaccine projects (March 2020-January 2021).**

**Table S5. OpenVaccine Donors.**
