# Supplementary material for: Theoretical basis for stabilizing messenger RNA through secondary structure design
Source: Nucleic Acids Res. 2021 Sep 14;49(18):10604–17. doi: 10.1093/nar/gkab764 (PMC8499941; doi:10.1093/nar/gkab764)
Supplement: gkab764_Supplemental_Files [file gkab764_supplemental_files.zip › SupportingInformation_29Jul2021_v1.pdf]

Supporting Information for "Theoretical basis for stabilizing messenger RNA through secondary structure design"

Appendix A: Derivation of AUP. To describe the degradation rate of an RNA molecule, we imagine that each nucleotide at position *i* has a rate of degradation $k_{cleavage}(i)$. To focus our presentation, we imagine that the degradation is due to inline hydrolysis, but the framework below generalizes to any degradation process that is suppressed by formation of RNA structure, including digestion by endonucleases for *in vivo* applications. The probability that the nucleotide backbone remains intact at nucleotide *i* after time *t* is

$$p_{intact}(i,t) \; = e^{-k_{cleavage}(i)\, t}. \tag{1}$$

The probability that the overall RNA with length *N* remains intact with no chain breaks after time *t* is then

$$p_{intact}{}^{overall}(t) = \prod_{i=1}^{N} p_{intact}(i,t) = \prod_{i=1}^{N} e^{-k_{cleavage}(i) t}$$
$$= \exp(-\textstyle\sum_{i=1}^{N} k_{cleavage}(i)\, t). \tag{2}$$

Here, we have assumed that the probability of cleavage at any given position is independent of cleavage events at other positions in the RNA. If this is not true, the expression will still remain correct at times when there are 0 or 1 cleavage events, and that is the time range most relevant for improving RNA stability. Given that assumption, eq. (2) gives an exactly exponential dependence of the overall degradation of the RNA with respect to time *t*:

$$\mathrm{p}_{\mathrm{intact}}^{overall}(t) = e^{-k_{cleavage}{}^{overall} t}, \tag{3}$$

with

$$k_{cleavage}{}^{overall} = \textstyle\sum_{i=1}^{N} k_{cleavage}(i). \tag{4}$$

The degradation half-life of the RNA is $t_{1/2} = \frac{\ln 2}{k_{cleavage}{}^{overall}}$.

We note that the above expression is analogous to modelling the rate of chemical modification at a given nucleotide. Models assuming first-order kinetics and two exponential distributions for paired and unpaired nucleotides have been used to incorporate structure probing data in structure prediction (1-3).

The rate at which an RNA is hydrolyzed at a specific location along its backbone $k_{cleavage}(i)$ depends on the ability of the phosphodiester bond to adopt the in-line attack conformation (Figure 1B), or more generally for the RNA to adopt a conformation that can be accessed by a degrading agent, like a protein nuclease. Here and below, we work out the consequences of a simple model reflecting the knowledge that in-line hydrolysis generally occurs at nucleotides that are unpaired but is strongly suppressed by pairing of the nucleotide into double-stranded segments of the secondary structure (4,5). Since RNA chains fluctuate between multiple secondary structures with a characteristic timescale of milliseconds (6), faster than the degradation rates discussed above, we write the overall cleavage rate as averaged over the equilibrated structural ensemble of the RNA,

$$k_{cleavage}{}^{overall} = \sum_{s\in\{S\}} p(s)\ \sum_i\ k_{cleavage}(i|s), \quad (5)$$

where {*S*} is the full set of structures that the RNA molecule is capable of adopting, and $p(s)$ is the probability of forming a structure *s*. The rate of cleavage $k_{cleavage}(i|s)$ at each position *i* within a structure *s* will, in general have a complex dependence on the sequence and structural context. For example, the cleavage rate will depend on whether the nucleotide is in a hairpin loop, where it is in within the loop, whether other loop nucleotides might promote in-line cleavage through acid-base catalysis, whether the loop has non-canonical pairs, etc. Without additional empirical knowledge, we assume that the cleavage rate for unpaired nucleotides can be approximated by a constant rate $k_{cleavage}{}^{unpaired}$ if nucleotide *i* is unpaired, and zero if paired. Then eq. (5) becomes:

$$k_{cleavage}{}^{overall} = \sum_{s\in\{S\}} \sum_i\ \ p(s)\ k_{cleavage}(i|s)$$

$$= \sum_i \sum_{s\in\{S\}} p(s) k_{cleavage}(i|s)$$

$$= \sum_i \sum_{s\in\{S\}} p(s) I(i \text{ unpaired in } s)\, {k_{cleavage}}^{unpaired}$$

$$= {k_{cleavage}}^{unpaired} \sum_i p_{unpaired}(i)$$

$$= {k_{cleavage}}^{unpaired} \times \text{SUP}, \qquad (6)$$

where $I(i \text{ unpaired in } s)$ is 1 if nucleotide *i* is unpaired in the structure *s* and 0 otherwise. In the last line of (6), we introduce the definition of sum of unpaired probabilities (SUP),

$$\text{SUP} = \sum_i p_{unpaired}(i). \qquad (7)$$

Overall, the total rate of cleavage may be approximated as this measure, the sum of unpaired probabilities across all nucleotides of the RNA, multiplied by a constant ${k_{cleavage}}^{unpaired}$ that reflects the average cleavage rate of an unpaired nucleotide.

It is important to point out that the total rate scales with the sum of the unpaired probabilities of the RNA's nucleotides – longer RNA molecules are expected to degrade faster in proportion to their length. This relation is better reflected by a rearrangement of (6) to:

$${k_{cleavage}}^{overall} = {k_{cleavage}}^{unpaired} \times N \times \text{AUP} \qquad (8)$$

where the average unpaired probability (AUP) is

$$\text{AUP} = \frac{1}{N} \sum_i p_{unpaired}(i) = \frac{1}{N} \text{SUP}. \qquad (9)$$

Appendix B. *Target identification for mRNA design and optimization.* Messenger RNAs encoding five target proteins were chosen for this work: 1) a candidate multi-epitope vaccine design (MEV) derived from SARS-CoV-2 spike and nucleocapsid proteins, 2) nanoluciferase, 3) eGFP with a degron sequence (eGFP+deg), 4) the receptor binding domain (RBD) of the SARS-CoV-2 spike protein, and 5) the full SARS-CoV-2 spike protein. Here we provide in detail the rationale behind how the constructs were chosen and designed.

Nanoluciferase and eGFP+degron were chosen for their ubiquitous use in biomedical research. The nanoluciferase sequence was taken from ref. (7). The choice of eGFP+degron allowed for close comparison with designed mRNAs from ref. (8), which investigated the relationships between mRNA structure, stability and translation using the eGFP+degron, while nanoluciferase was chosen to complement another easily assayed, commonly used model protein in mammalian systems.

For the MEV protein, we chose peptide sequences from SARS-CoV with verified T cell-positive assays that were identical in SARS-CoV-2, and one that maximized population coverage according to MHC allele mapping(9). Two of the epitopes (GYQPYRVVVL and PYRVVVLSF) map directly onto the receptor binding domain of the S (spike) protein, which we hypothesized to be important in eliciting the proper antibody/immune response. The last epitope (LSPRWYFYY), from the N protein, was chosen for its potentially high coverage of effectiveness in the global population. To test our hypothesis against a wide range of mRNA lengths, we chose to include only three peptide epitopes to introduce a shorter mRNA in our MEV design, but it should be noted that a more realistic MEV may feature many more epitopes (10,11).

The SARS-CoV-2 spike RBD is derived from the reported structure of the spike protein in the pre-fusion conformation(12). Similarly, the protein chosen for the "JEV + spike" protein is not the full sequence as found in the genome of SARS-CoV-2(13), but as reported in the pre-fusion conformation, which is hypothesized to promote an enhanced immune response in use as a vaccine. For three of the proteins

(nanoluciferase, RBD, and “JEV+ spike” protein), the signal sequence from the Japanese encephalitis virus (JEV) was added to the N-terminus to encode designs for a secreted vaccine (14). The “JEV + spike” protein additionally had StrepII tag appended.

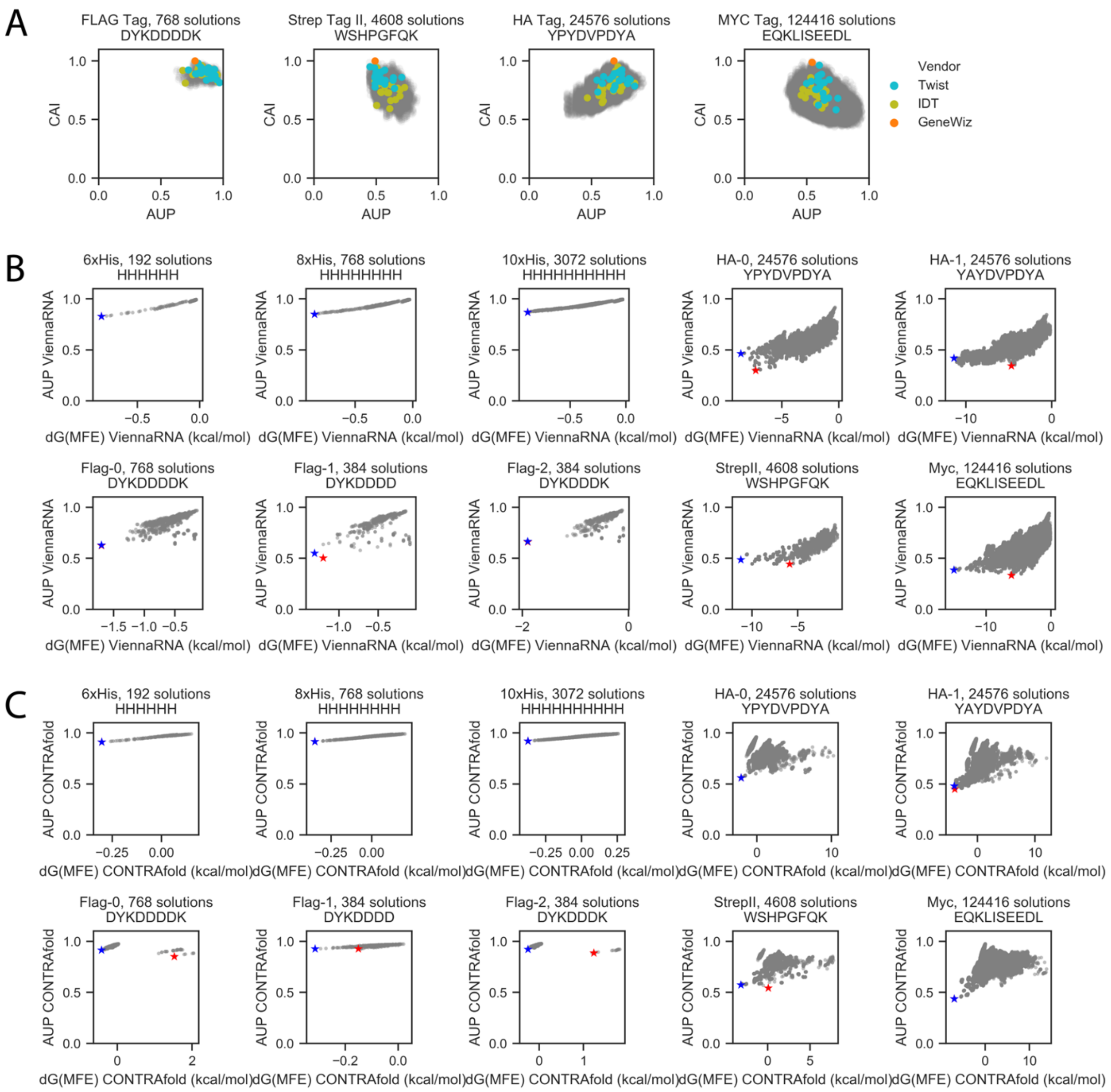

Figure S1. (A) CAI vs. AUP for mRNAs encoding peptide tags in main text. (B) dG(MFE) vs. AUP, predicted in ViennaRNA and (C) CONTRAfold for all small tag protein systems studied by enumerating coding mRNAs. Blue star indicates solution with lowest dG(MFE), red star indicates solution with lowest AUP.

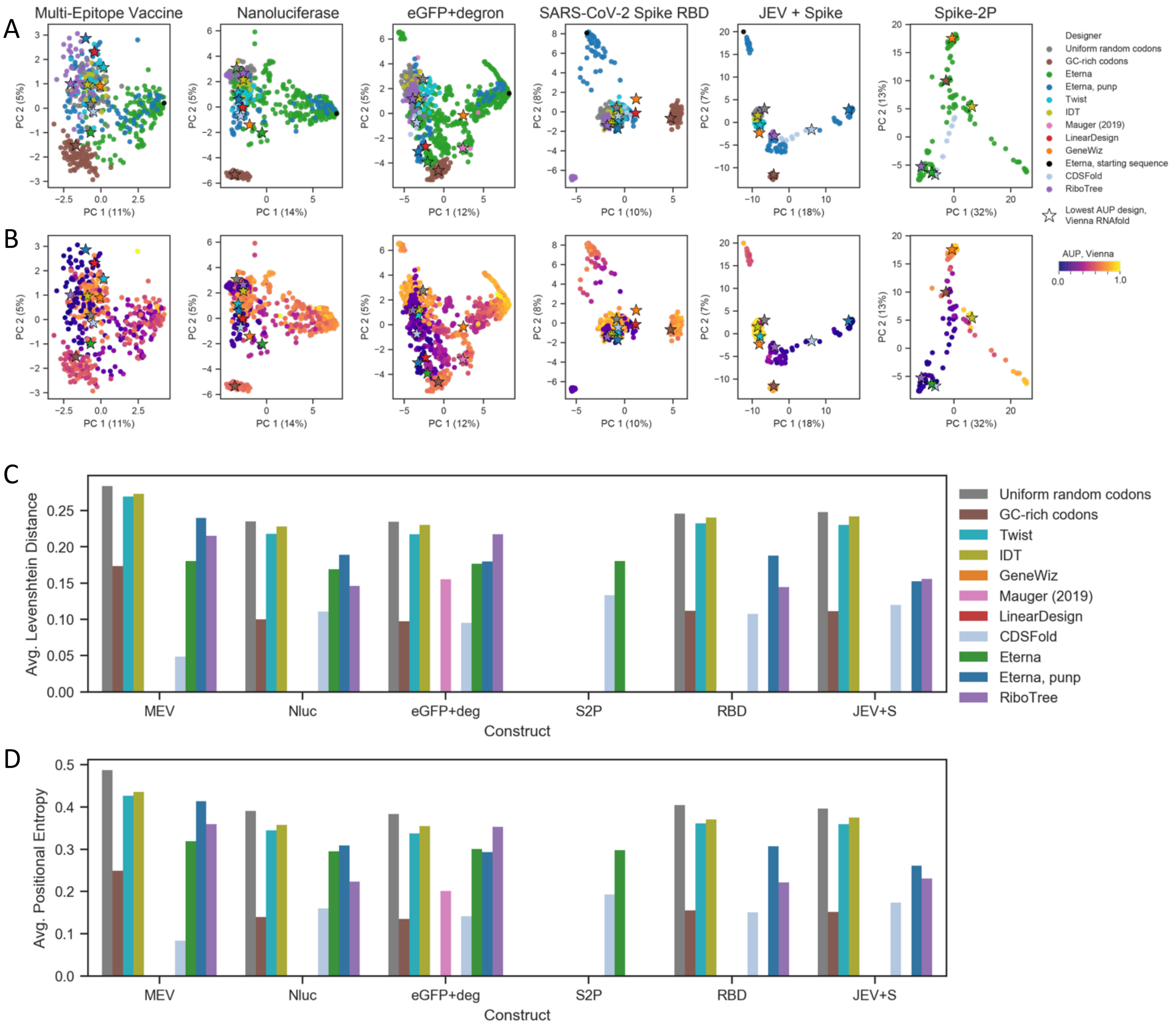

Figure S2. (A) Eterna designs encompass a diverse sequence space when compared to mRNA sequences generated by random sampling, visualized with principal component analysis. (B) The same visualization, colored by AUP as calculated in ViennaRNA. (C) Average pairwise Levenshtein distance and (D) average positional entropy between sequences of different classes.

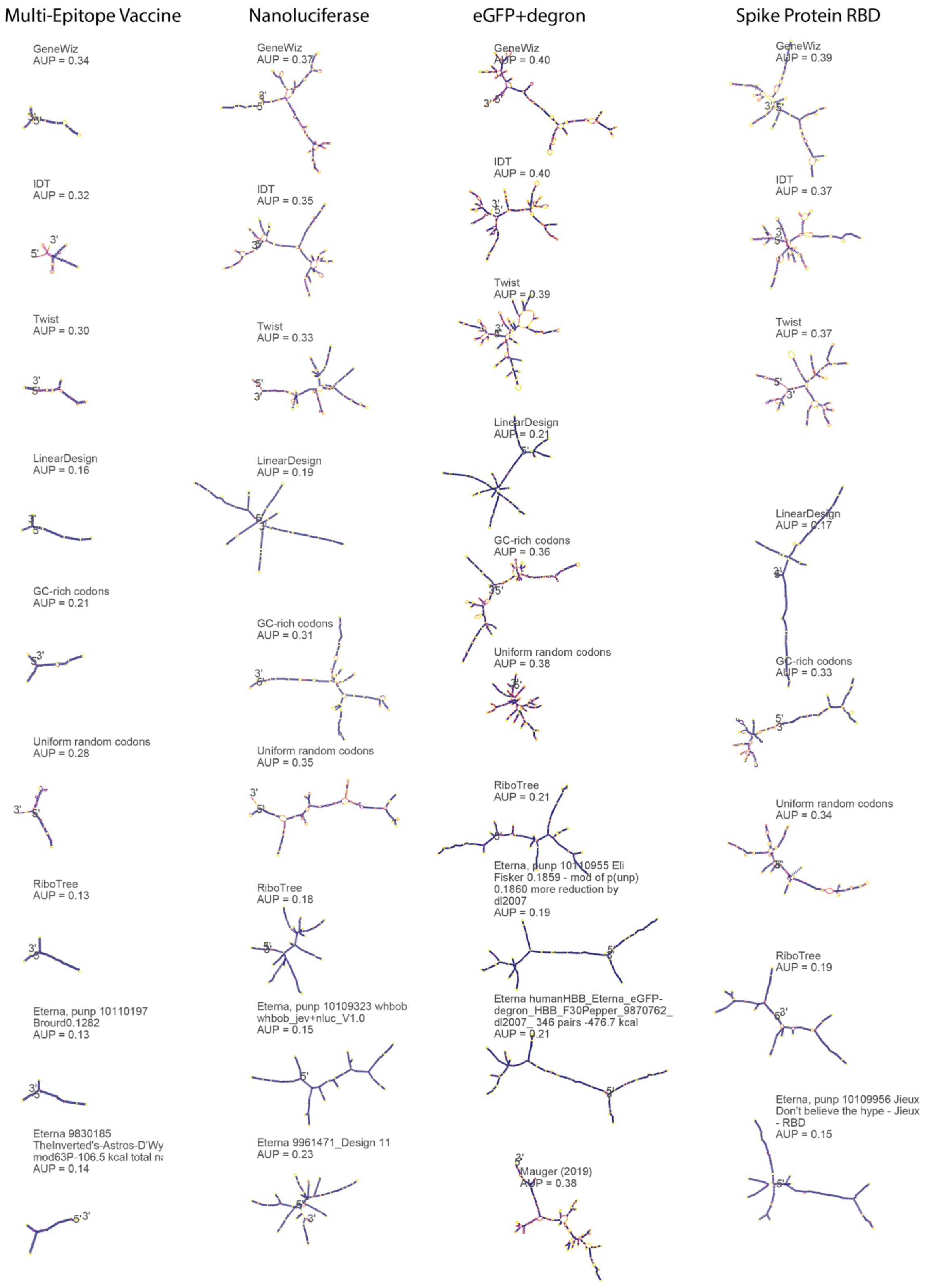

Figure S3: MFE structures of minimum AUP solutions from each design method, predicted with ViennaRNA, for each of the four "p(unp) challenges" mRNA design challenges.

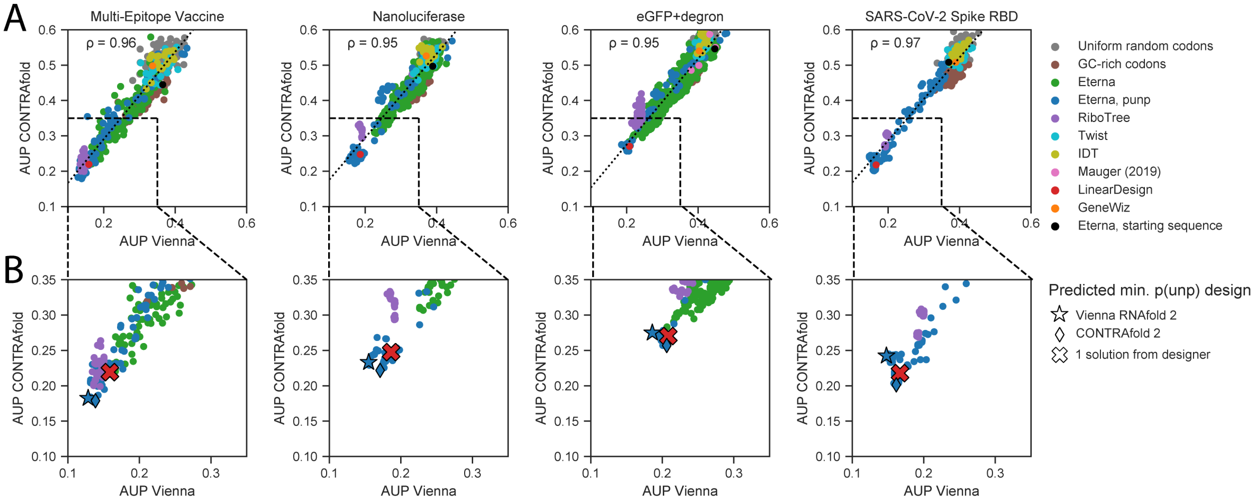

Figure S4. For the four mRNA design challenges, using different secondary structure packages to calculate AUP results in minimum AUP sequences with slightly different AUP values. (A) AUP, as calculated in Vienna RNAfold and CONTRAfold, have high correlation, ranging from Pearson R = 0.95 to 0.97 for these constructs. (B) Inset of (A) to better view low AUP sequences. For the Nluc, eGFP+deg, and spike RBD design challenges, the predicted min. AUP sequences differ.

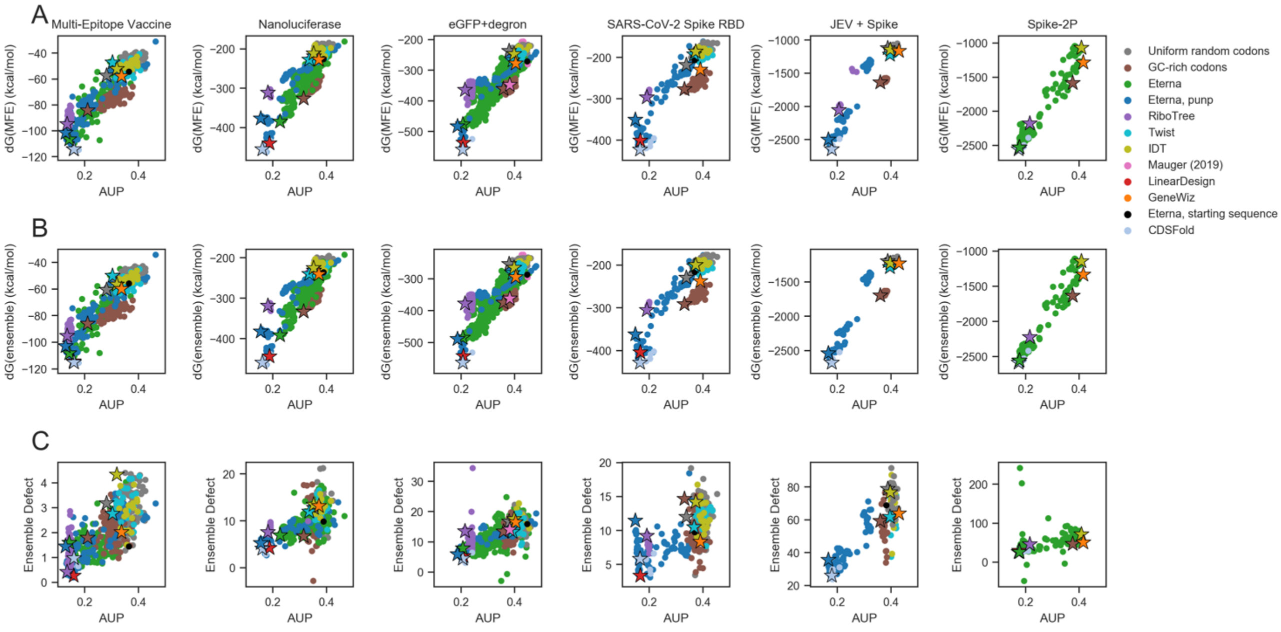

Figure S5. Comparing (A) AUP vs. ΔG(MFE) (top row), (B) AUP vs. ΔG(ensemble) (bottom row), and (C) AUP vs. (ΔG(MFE) – ΔG(ensemble)) for all mRNA design challenges demonstrates similar trends between ΔG(MFE) and ΔG(ensemble) in comparing to AUP.

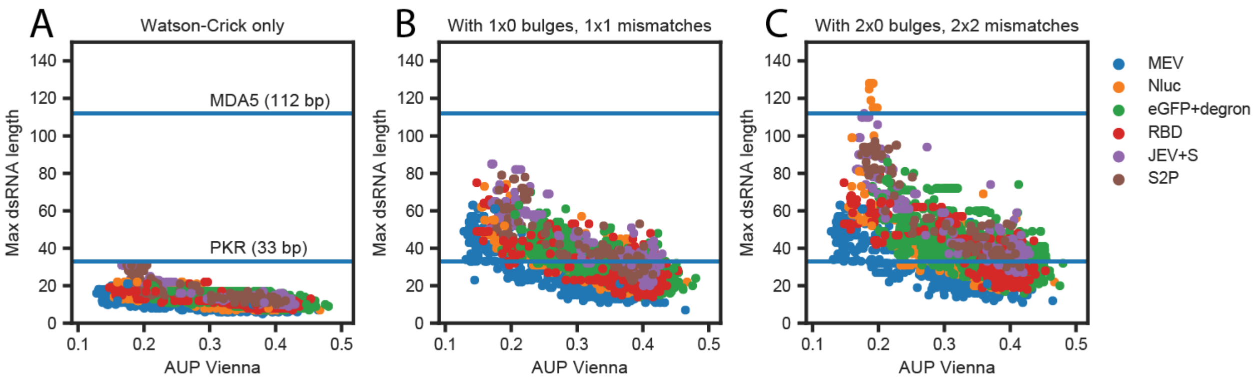

Figure S6. Considering dsRNA lengths with 0 mismatches (A), 1x0 bulges or 1x1 mismatches (B), or mismatches up to size 2x2 or bulges up to size 2x0 (C) indicates that if dsRNA-binding proteins bind bulges or mismatches with similar affinity, RNAs may need to be redesigned to have maximum dsRNA lengths less than 33 bp to avoid, i.e., PKR recognition.

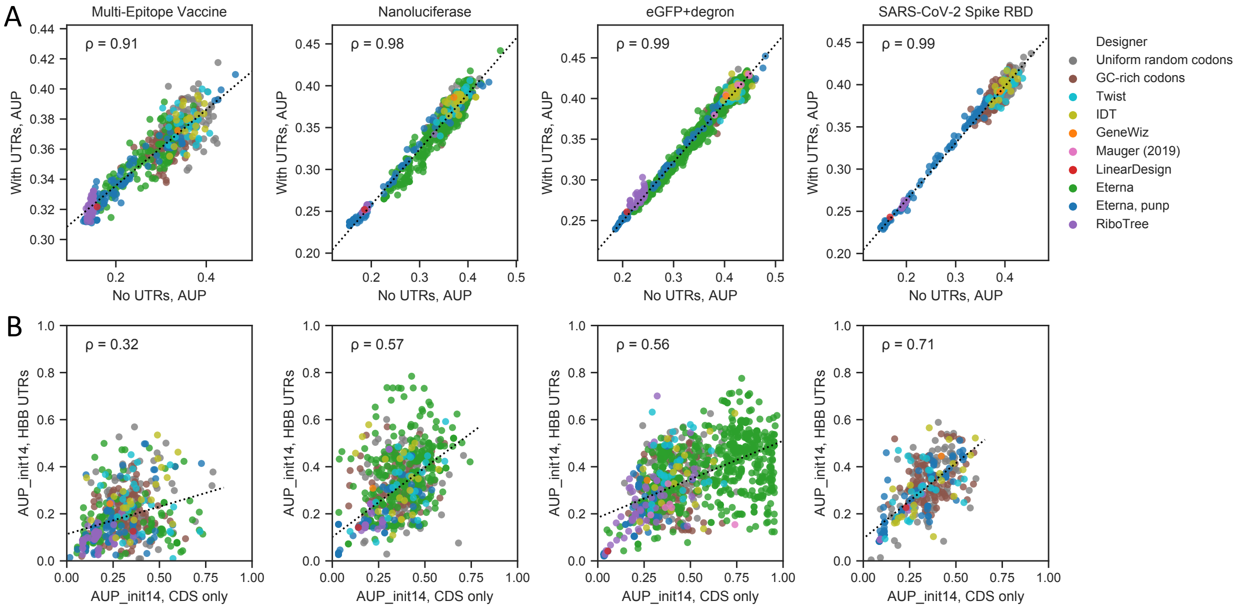

Figure S7. AUP of longer mRNAs is less affected by including constant untranslated regions (UTRs). (A) AUP in the absence (x-axis) and presence (y-axis) of HBB 5' 3' UTRs are highly correlated. (B) The unpaired probability of the first 14 nucleotides is weakly correlated in the absence (x-axis) and presence of UTRs.

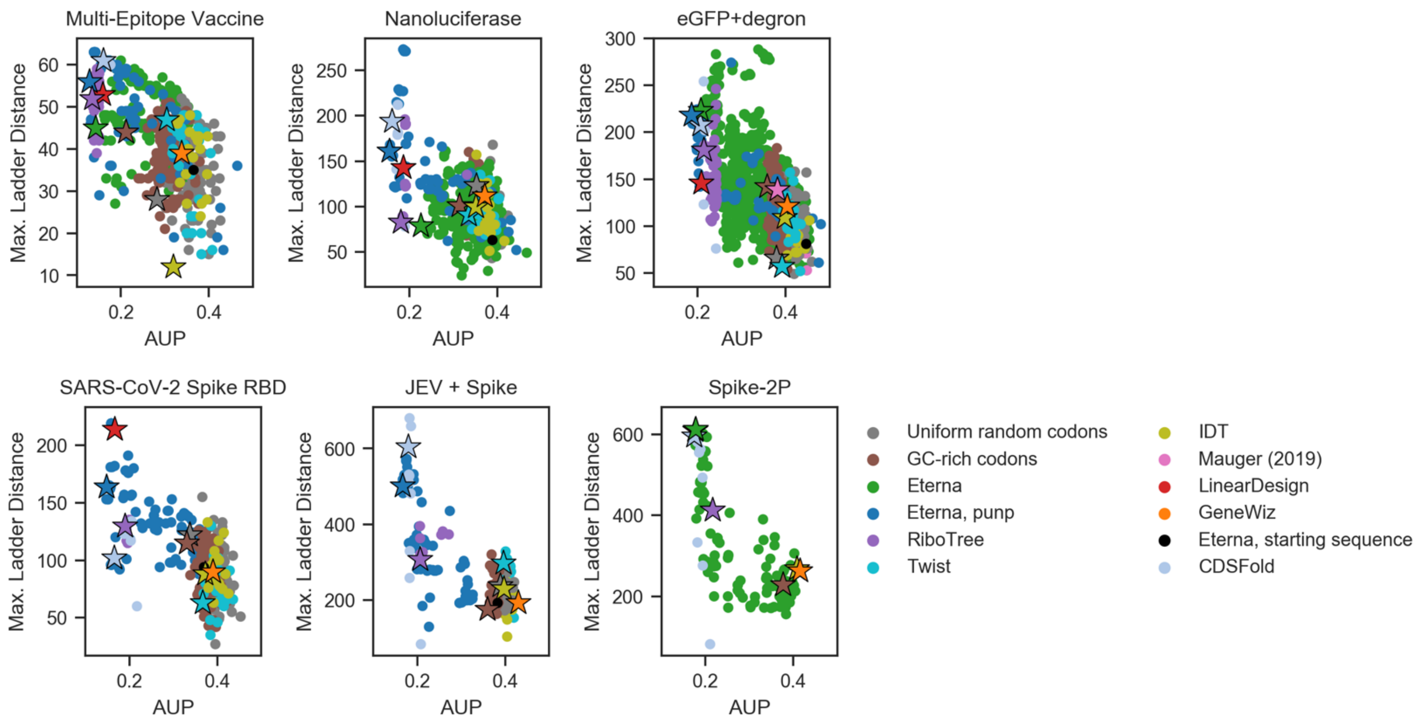

Figure S8. Maximum Ladder distance vs. AUP for all challenges.

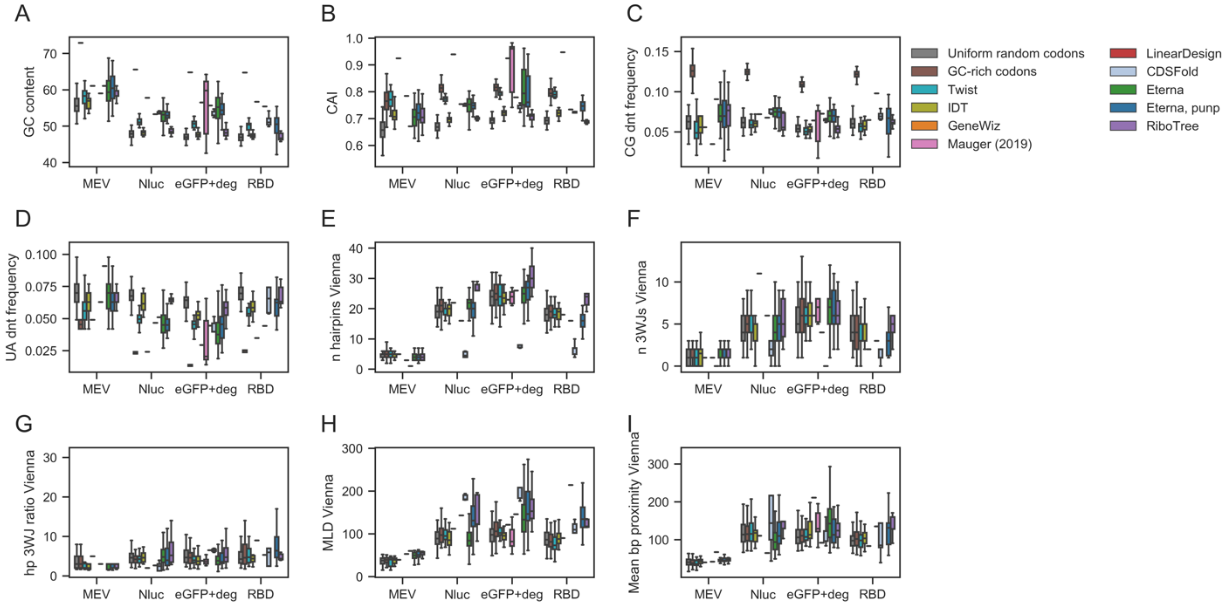

Figure S9. Summary statistics for sequence- and structure-based metrics calculated for the compared "p(unp) challenges" sequences, calculated in ViennaRNA. Box represents 25th to 75th quartile, whiskers represent 1.5 * inter-quartile range.

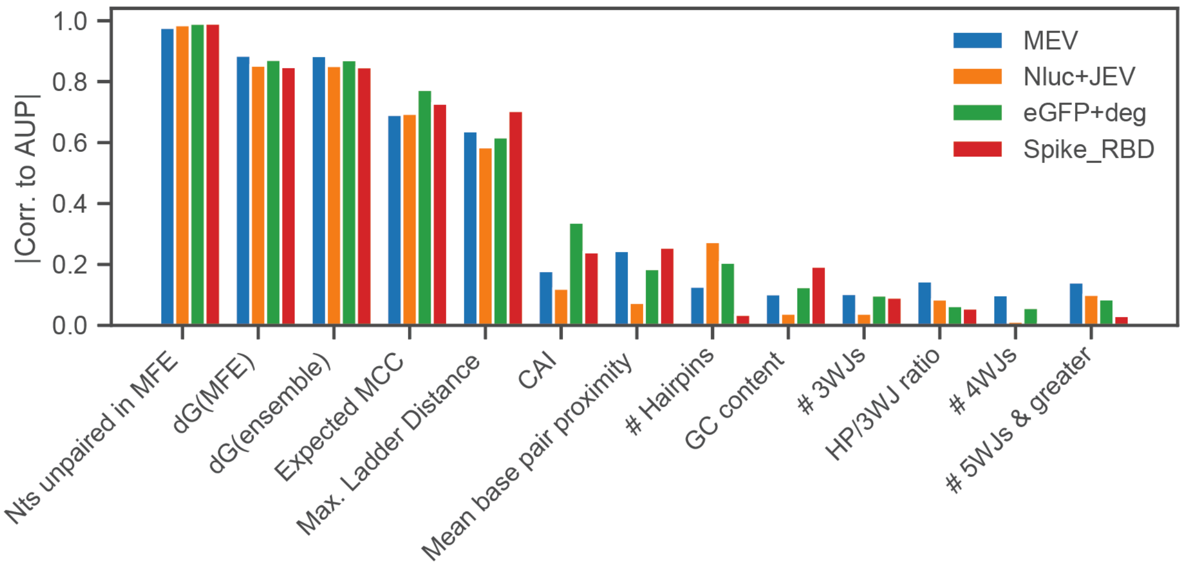

Figure S10. Correlation of tested measures (calculated in ViennaRNA prediction package) to AUP for all mRNA design challenges.

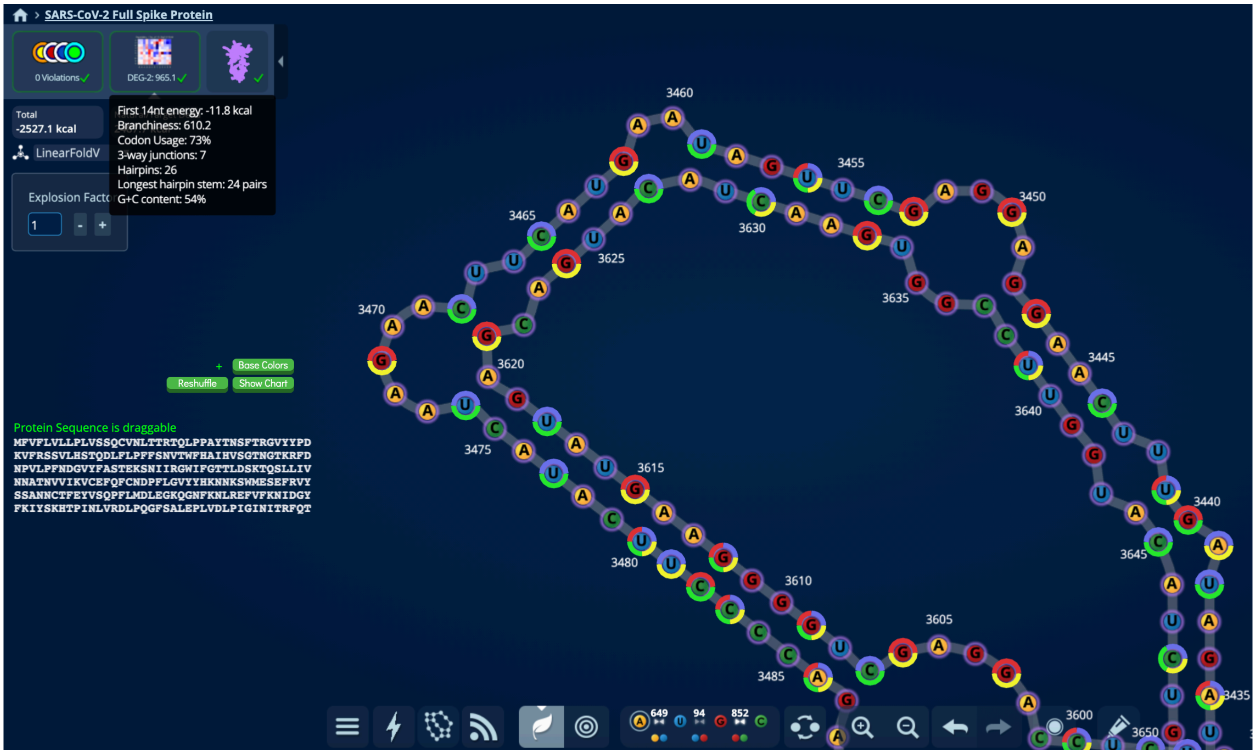

Figure S11. Screenshot of calculated properties available to Eterna participants during design of full-length spike protein mRNAs.

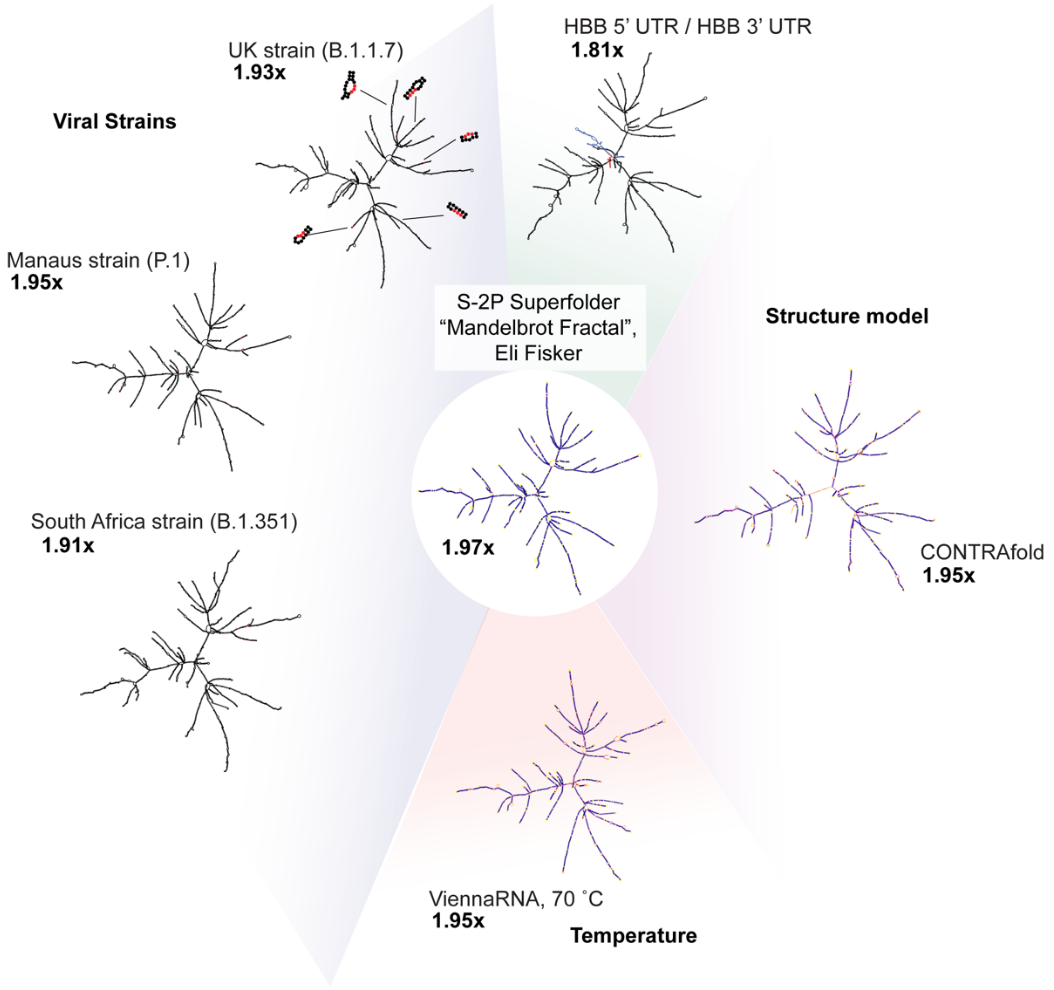

Figure S12. Stabilization derived from a highly branched, low AUP solution is robust to small variations in protein sequence, variations in untranslated region (UTR), choice of folding algorithm, and higher temperatures. Compare to main text Fig. 6, which is based on a distinct, less branched AUP solution that shows analogous robustness to these changes.

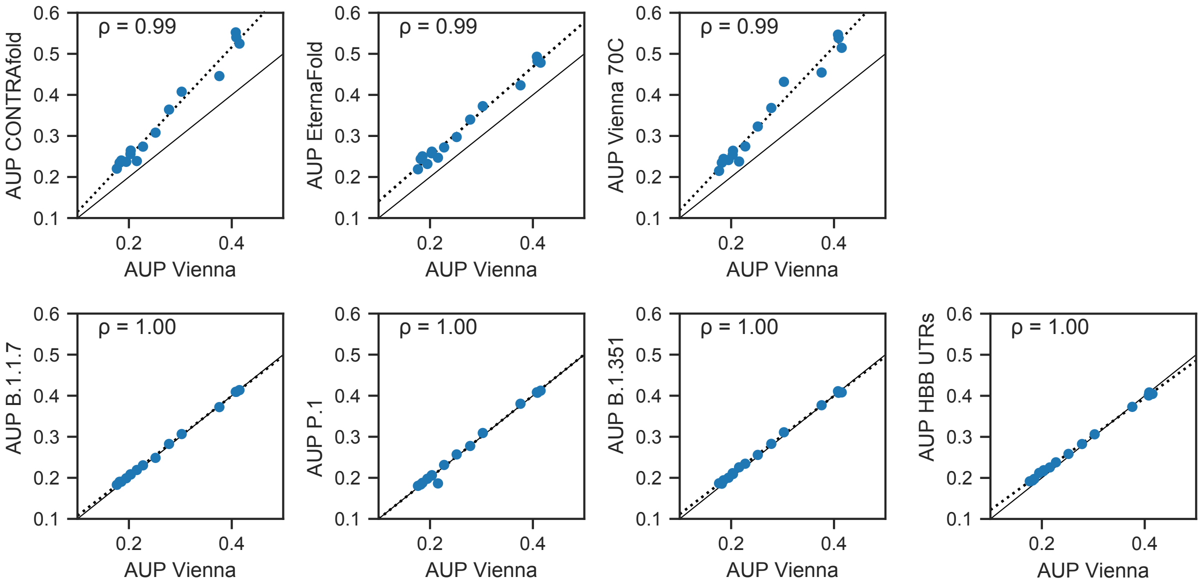

Figure S13. For an example series of S-2P constructs, AUP of original designed CDS in Vienna is highly correlated with predicted AUP in other packages, with the sequence patched for other strains, and in the context of UTRs.

References

1. Cordero, P. and Das, R. (2015) Rich RNA Structure Landscapes Revealed by Mutate-and-Map Analysis. *PLoS Comput Biol*, **11**, e1004473.
2. Washietl, S., Hofacker, I.L., Stadler, P.F. and Kellis, M. (2012) RNA folding with soft constraints: reconciliation of probing data and thermodynamic secondary structure prediction. *Nucleic Acids Res.*, **40**, 4261-4272.
3. Zarringhalam, K., Meyer, M.M., Dotu, I., Chuang, J.H. and Clote, P. (2012) Integrating chemical footprinting data into RNA secondary structure prediction. *PLoS One*, **7**, e45160.
4. Mikkola, S., Kaukinen, U. and Lonnberg, H. (2001) The effect of secondary structure on cleavage of the phosphodiester bonds of RNA. *Cell Biochem Biophys*, **34**, 95-119.
5. Regulski, E.E. and Breaker, R.R. (2008) In-line probing analysis of riboswitches. *Methods Mol Biol*, **419**, 53-67.
6. Chen, S.-J. (2008) RNA folding: conformational statistics, folding kinetics, and ion electrostatics. *Annual review of biophysics*, **37**, 197-214.
7. Leppek, K., Fujii, K., Quade, N., Susanto, T.T., Boehringer, D., Lenarčič, T., Xue, S., Genuth, N.R., Ban, N. and Barna, M. (2020) Gene- and Species-Specific Hox mRNA Translation by Ribosome Expansion Segments. *Molecular Cell*, **80**, 980-995.e913.
8. Mauger, D.M., Cabral, B.J., Presnyak, V., Su, S.V., Reid, D.W., Goodman, B., Link, K., Khatwani, N., Reynders, J., Moore, M.J. *et al.* (2019) mRNA structure regulates protein expression through changes in functional half-life. *Proc Natl Acad Sci U S A*, **116**, 24075-24083.

9. Ahmed, S.F., Quadeer, A.A. and McKay, M.R. (2020) Preliminary Identification of Potential Vaccine Targets for the COVID-19 Coronavirus (SARS-CoV-2) Based on SARS-CoV Immunological Studies. *Viruses*, **12**, 254.
10. Tahir Ul Qamar, M., Rehman, A., Tusleem, K., Ashfaq, U.A., Qasim, M., Zhu, X., Fatima, I., Shahid, F. and Chen, L.L. (2020) Designing of a next generation multiepitope based vaccine (MEV) against SARS-COV-2: Immunoinformatics and in silico approaches. *PLoS One*, **15**, e0244176.
11. Prachar, M., Justesen, S., Steen-Jensen, D.B., Thorgrimsen, S., Jurgons, E., Winther, O. and Bagger, F.O. (2020) Identification and validation of 174 COVID-19 vaccine candidate epitopes reveals low performance of common epitope prediction tools. *Sci Rep*, **10**, 20465.
12. Wrapp, D., Wang, N., Corbett, K.S., Goldsmith, J.A., Hsieh, C.-L., Abiona, O., Graham, B.S. and McLellan, J.S. (2020) Cryo-EM structure of the 2019-nCoV spike in the prefusion conformation. *Science*, **367**, 1260-1263.
13. Wu, F., Zhao, S., Yu, B., Chen, Y.M., Wang, W., Song, Z.G., Hu, Y., Tao, Z.W., Tian, J.H., Pei, Y.Y. *et al.* (2020) A New Coronavirus Associated With Human Respiratory Disease in China. *Nature*, **579**, 265-269.
14. Liu, H., Wu, R., Yuan, L., Tian, G., Huang, X., Wen, Y., Ma, X., Huang, Y., Yan, Q., Zhao, Q. *et al.* (2017) Introducing a Cleavable Signal Peptide Enhances the Packaging Efficiency of Lentiviral Vectors Pseudotyped With Japanese Encephalitis Virus Envelope Proteins. *Virus research*, **229**, 9-16.
